# Physically informed artificial neural networks for atomistic modeling of materials

**DOI:** 10.1038/s41467-019-10343-5

**Published:** 2019-05-28

**Authors:** G. P. Purja Pun, R. Batra, R. Ramprasad, Y. Mishin

**Affiliations:** 1 Department of Physics and Astronomy, MSN 3F3, George Mason University, Fairfax, VA 22030 USA; 2 School of Materials Science and Engineering, Georgia Institute of Technology, Atlanta, GA 30332 USA

## Abstract

Large-scale atomistic computer simulations of materials heavily rely on interatomic potentials predicting the energy and Newtonian forces on atoms. Traditional interatomic potentials are based on physical intuition but contain few adjustable parameters and are usually not accurate. The emerging machine-learning (ML) potentials achieve highly accurate interpolation within a large DFT database but, being purely mathematical constructions, suffer from poor transferability to unknown structures. We propose a new approach that can drastically improve the transferability of ML potentials by informing them of the physical nature of interatomic bonding. This is achieved by combining a rather general physics-based model (analytical bond-order potential) with a neural-network regression. This approach, called the physically informed neural network (PINN) potential, is demonstrated by developing a general-purpose PINN potential for Al. We suggest that the development of physics-based ML potentials is the most effective way forward in the field of atomistic simulations.

## Abstract (web summary)

Traditional machine learning potentials suffer from poor transferability to unknown structures. Here the authors present an approach to improve the transferability of machine-learning potentials by including information on the physical nature of interatomic bonding.

## Introduction

Large-scale molecular dynamics (MD) and Monte Carlo (MC) simulations of materials are traditionally implemented using classical interatomic potentials predicting the potential energy and Newtonian forces acting on atoms. Computations with such potentials are very fast and afford access to systems with millions of atoms and MD simulation times up to hundreds of nanoseconds. Such simulations span a wide range of time and length scales and constitute a critical component of the multiscale approach in materials modeling and computational design.

Several functional forms of interatomic potentials have been developed over the years, including the embedded-atom method (EAM)^1–3^, the modified EAM (MEAM)^4^, the angular-dependent potentials^5^, the charge-optimized many-body potentials^6^, reactive bond-order potentials^7–9^, and reactive force fields^10^ to name a few. These potentials address particular classes of materials or particular types of applications. Their functional forms depend on the physical and chemical models chosen to describe interatomic bonding in the respective class of materials.

**Fig. 1:**
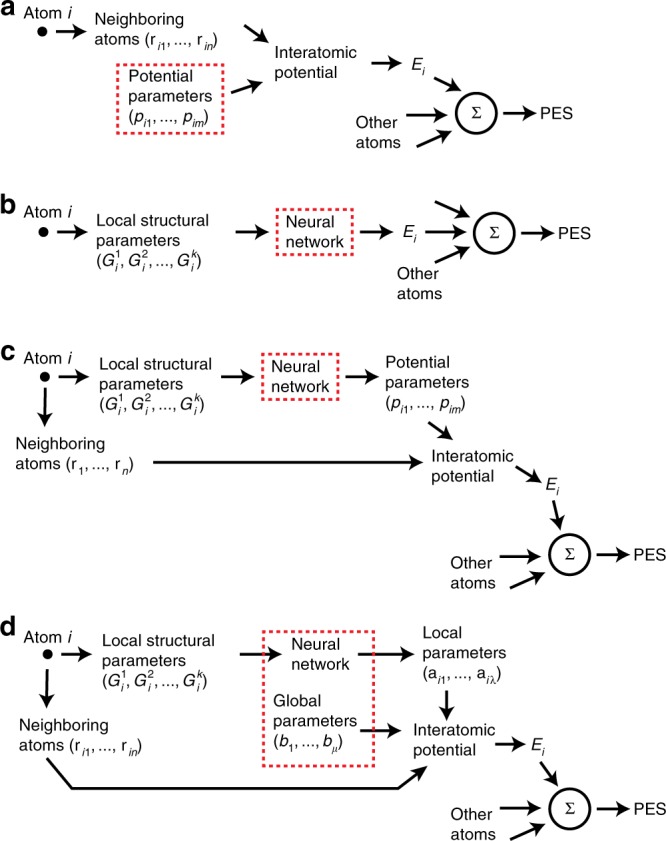
Flowcharts of the development of atomistic potentials. **a** Traditional interatomic potential. **b** Mathematical NN potential. **c** Physically informed NN (PINN) potential with all-local parameters. **d** PINN potential with parameters divided into local and global. The dashed rectangle outlines the objects requiring parameter optimization. PES is the potential energy surface of the material

A common feature of all traditional potentials is that they express the potential energy surface (PES) of the system, *E* = *E*(**r**_1_, ..., **r**_*N*_, **p**), as a relatively simple function of atomic coordinates (**r**_1_, ..., **r**_*N*_), *N* being the number of atoms (Fig. 1a). Knowing the PES, the forces acting on the atoms can be computed by differentiation and used in MD simulations. The potential functions depend on a relatively small number of fitting parameters **p** = (*p*_1_, ..., *p*_*m*_) (typically, *m* = 10–20) and are optimized (trained) on a relatively small database of experimental data and first-principles density functional theory (DFT) calculations. The traditional potentials are, of course, much less accurate than DFT calculations. Nevertheless, many of them demonstrate a reasonably good transferability to atomic configurations lying well outside the training dataset. This important feature owes its origin to the incorporation of at least some basic physics in the potential form. As long as the nature of chemical bonding remains the same as assumed during the potential development, the potential can predict the system energy adequately even for new configurations not seen during the training process. Unfortunately, the construction of good quality potentials is a long and painful process requiring personal experience and intuition and is more art than science^8,11^. In addition, the traditional potentials are specific to a particular class of materials and cannot be easily extended to other materials or improved in a systematic manner.

During the past decade, a new direction has emerged wherein interatomic potentials are developed by employing machine-learning (ML) methods^12–22^. The idea was originally conceived in the chemistry community in the 1990s in the effort to improve the accuracy of inter-molecular force fields^23,24^, an approach that was later adopted by the physics and materials science communities. The general idea is to forego the physical insights and reproduce the PES by interpolating between DFT data points using high-dimensional nonlinear regression methods such as the Gaussian process regression^19,25–27^, interpolating moving least squares^28^, kernel ridge regression^12,20,21^, compressed sensing^29,30^, gradient-domain machine-learning model^31^, or the artificial neural network (NN) approach^13–18,32–38^. If properly trained, a ML potential can predict the system energy with a nearly DFT accuracy (a few meV/atom). ML potentials are not specific to a particular class of materials or type of chemical bonding. They can be improved systematically if weaknesses are discovered or new DFT data become available. The training process can be implemented on-the-fly by running ab initio MD simulations^26^.

A major weakness of ML potentials is their poor transferability. Being purely mathematical constructions devoid of any physical meaning, they can accurately interpolate the energy between the training configurations but are generally incapable of properly extrapolating the energy to unknown atomic environments. As a result, the performance of ML potentials outside the training domain can be very poor. There is no reason why a purely mathematical extrapolation scheme would deliver physically meaningful results outside the training database. This explains why the existing ML potentials are usually (with rare exceptions^39^) narrowly focused on, and only tested for, a particular type of physical properties. This distinguishes them from the traditional potentials which, although less accurate, are designed for a much wider range of applications and diverse properties.

In this work we propose a new approach that can drastically improve the transferability of ML potentials by informing them of the physical nature of interatomic bonding. We focus on NN potentials as an example, but the approach is general and can be readily extended to other methods of nonlinear regression. Like all ML potentials, the proposed physically informed NN (PINN) potentials are trained using a large DFT dataset. However, by contrast to the existing, mathematical NN potentials, the PINN potentials incorporate the basic physics and chemistry of atomic interactions leveraged by the extraordinary adaptivity and trainability of NNs. The PINN potentials thus strike a golden compromise between the two extremes represented by the traditional, physics-guided interatomic potentials, and the mathematical NN potentials.

The general idea of combining traditional interatomic potentials with NNs was previously discussed by Malshe et al.^40^, who constructed an adjustable Tersoff potential^41–43^ for a Si_5_ cluster. Other authors have also applied machine-learning methods to parameterize physics-based models of molecular interactions, primarily in the context of broad exploration of the compositional space of molecular (mostly organic) matter^44–46^. Glielmo et al.^47^ recently proposed to construct *n*-body Gaussian process kernels to capture the *n*-body nature of atomic interactions in physical systems. The PINN potentials proposed in this paper are inspired by such approaches but extend them to (1) more advanced physical models with a broad applicability, and (2) large-scale systems by introducing local energies *E*_*i*_ linked to local structural parameters $G_i^l$. The focus is placed on the exploration of the configurational space of defected solids and liquids in single-component and, in the future, binary or multicomponent systems. The main goal is to improve the transferability of interatomic potentials to unknown atomic environments while keeping the same level of accuracy of training as normally achieved with mathematical machine-learning potentials.

## Results

### Physically informed neural network potentials

The currently existing, mathematical NN potentials^13–18,32–36^ partition the total energy *E* into a sum of atomic energies, $E = \mathop {\sum}\nolimits_i {E_i}$. A single NN is constructed to express each atomic energy *E*_*i*_ as a function of a set of local fingerprint parameters (also called symmetry parameters^13^) $(G_i^1,G_i^2,...,G_i^k)$. These parameters encode the local environments of the atoms. The network is trained by minimizing the error between the energies predicted by the NN and the respective DFT total energies for a large set of atomic configurations. The flowchart of the method is depicted in Fig. 1b.

The proposed PINN model is based on the following considerations. A traditional, physics-based potential can always be trained to reproduce the energy of any given atomic configuration with any desired accuracy. Of course, this potential will not work well for other configurations. Imagine, however, that the potential parameters have been trained for a large set of reference structures, one structure at a time, each time producing a different parameter set **p**. Suppose that, during the subsequent simulations, we have a way of identifying, on the fly, a reference structure closest to any current atomic configuration. Then the accuracy of the simulation can be drastically improved by dynamically choosing the best set of potential parameters for every atomic configuration accoutered during the simulation. Now, since the atomic energy *E*_*i*_ only depends on the local environment of atom *i*, the best parameter set for computing *E*_*i*_ can be chosen by only examining the local environment of this atom. The energies of different atoms are then computed by using different, environment-dependent, parameter sets while keeping the same, physics-motivated functional form of the potential.

Instead of generating and storing a large set of discrete reference structures, we can construct a continuous NN-based function mapping the local environment of every atom on a parameter set of the interatomic potential optimized for that particular environment. Specifically, the local structural parameters (fingerprints) $G_i^l$ (*l* = 1, ..., *k*) of every atom *i* are fed into the network, which then maps them to the optimized parameter set **p**_*i*_ appropriate for atom *i*. Mathematically, the local energy takes the functional form

1 $$E_i = E_i\left( {{\mathbf{r}}_{i1},...,{\mathbf{r}}_{in},{\mathbf{p}}_i\left( {G_i^l({\mathbf{r}}_{i1},...,{\mathbf{r}}_{in})} \right)} \right),$$

where (**r**_*i*1_, ..., **r**_*in*_) are atomic positions in the vicinity of atom *i*.

In comparison with the direct mapping $G_i^l \mapsto E_i$ implemented by the mathematical NN potentials, we have added an intermediate step: $G_i^l \mapsto {\mathbf{p}}_i \mapsto E_i$. The first step is executed by the NN and the second by a physics-based interatomic potential. A flowchart of the two-step mapping is shown in Fig. 1c. It is important to emphasize that this intermediate step does not degrade the accuracy relative to the direct mapping, because a feedforward NN can always be trained to execute any real-valued function^48,49^. Thus, for any functional form of the potential, the NN can always adjust its architecture, weights and biases to achieve the same mapping as in the direct method. However, since the chosen potential form captures the essential physics of atomic interactions, the proposed PINN potential will display a better transferability to new atomic environments. Even if the potential parameters predicted by the NN for an unknown environment are not very accurate, the physics-motivated functional form will ensure that the results remain at least physically meaningful. This physics-guided extrapolation is likely to be more reliable than the purely mathematical extrapolation inherent in the existing NN potentials. Obviously, the same reasoning applies to the interpolation process as well, which can also be more accurate.

The functional form of the PINN potential must be general enough to be applicable across different classes of materials. In this paper we chose a simple analytical bond-order potential (BOP)^50–52^ that must work equally well for both covalent and metallic materials. For a single-component system, the BOP functions are specified in the Methods section. They capture the physical and chemical effects such as the pairwise repulsion between atoms, the angular dependence of the chemical bond strength, the bond-order effect (the more neighbors, the weaker the bond), and the screening of chemical bonds by surrounding atoms. In addition to being appropriate for covalent bonding, the proposed BOP form reduces to the EAM formalism in the limit of metallic bonding.

### Example: PINN potential for Al

To demonstrate the PINN method, we have constructed a general-purpose potential for aluminum. The training and validation datasets were randomly selected from a pre-existing DFT database^20,21^. Some additional DFT calculations have also been performed using the same methodology as in refs. ^20,21^. The selected DFT supercells represent seven crystal structures for a large set of atomic volumes under isotropic tension and compression, several slabs with different surface orientations, including surfaces with adatoms, a supercell with a single vacancy, five different symmetrical tilt grain boundaries, and an unrelaxed intrinsic stacking fault on the (111) plane with different translational states along the [211] direction. The database also includes several isolated clusters with the number of atoms ranging from 2 (dimer) to 79. The ground-state face centered cubic (FCC) structure was additionally subject to uniaxial tension and compression in the [100] and [111] directions at 0 K temperature. Most of the atomic configurations were snapshots of DFT MD simulations in the microcanonical (NVE) or canonical (NVT or NPT) ensembles for several atomic volumes at several temperatures. Some of the high-temperature configurations were part-liquid, part crystalline. In total, the database contains 3649 supercells (127592 atoms). More detailed information about the database can be found in the Supplementary Tables 1 and 2. To avoid overfitting or selection bias, the 10-fold cross-validation method was used during the training. The database was randomly partitioned in 10 subsets. One of them was set aside for validation and the remaining data was used for training. The process repeated 10 times for different choices of the validation subset.

The local structural parameters $G_i^l$ chosen for Al are specified in the Methods section. The NN contained two hidden layers with the same number of nodes in each. This number was increased until the training process produced a PINN potential with the root-mean-square error (RMSE) of training and validation close to 3–4 meV per atom, which was set as our goal. This is the level of accuracy of the DFT energies included in the database. For comparison, a mathematical NN potential was constructed using the same methodology. The number of hidden nodes of the NN was adjusted to give about the same number of fitted parameters and to achieve approximately the same RMSE of training and validation as for the PINN potential. Table 1 summarizes the training and validation errors averaged over the 10 cross-validation runs. One PINN and one NN potential were selected for a more detailed examination reported below.

**Table 1:** Fitting and validation errors of the straight NN and PINN models

Model	NN architecture	Number of parameters	RMSE of training (meV per atom)	RMSE of validation (meV per atom)
NN	60 × 16 × 16 × 1	1265	3.36	3.85
NN′	47 × 18 × 18 × 1	1225	3.62	3.54
PINN	60 × 15 × 15 × 8	1283	3.46	3.59

**Fig. 2:**
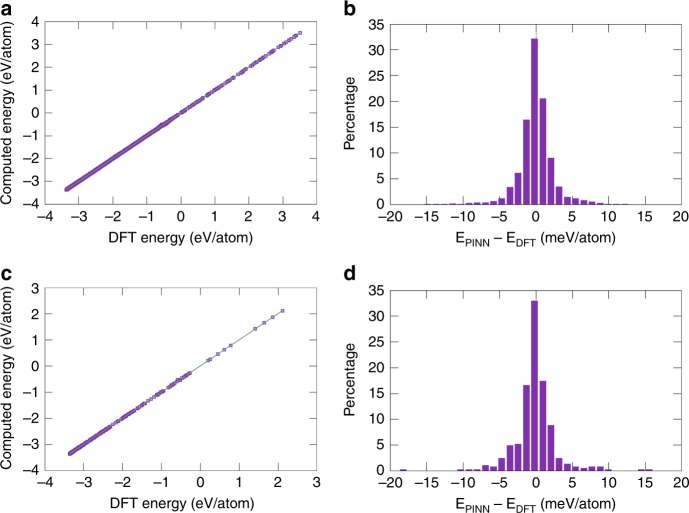
Accuracy of the PINN potential. **a**, **c** Energies of atomic configurations in the **a** training and **c** validation datasets computed with the PINN potential versus DFT energies. The straight line represents the perfect fit. **b**, **d** Error distributions in the **b** training and **d** validation datasets

Figure 2 and Supplementary Fig. 1 demonstrate excellent correlation between the predicted and DFT energies over a 7 eV per atom wide energy range for both potentials. The error distribution has a near-Gaussian shape centered at zero. Examination of errors in individual groups of structures (Supplementary Fig. 2) shows that the largest errors originate from the crystal structures (especially FCC, HCP, and simple hexagonal) subjected to large expansion.

**Fig. 3:**
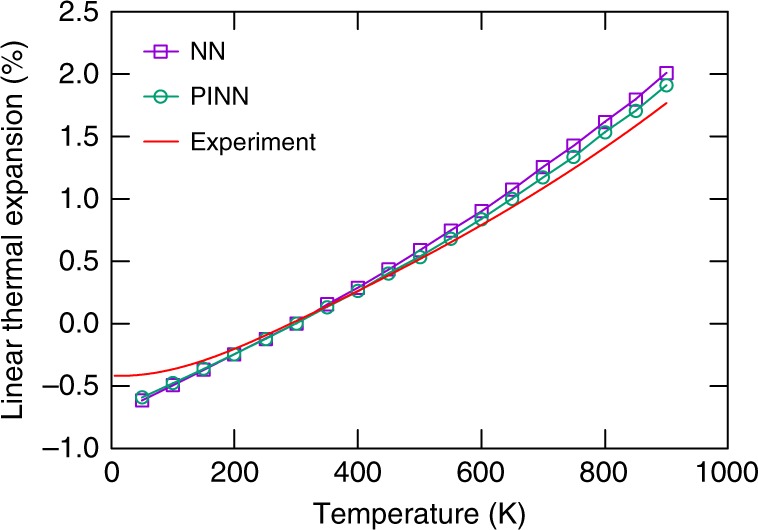
Linear thermal expansion of Al relative to room temperature (295 K) predicted by the PINN and NN potentials in comparison with experiment^60^

Table 2 summarizes some of the physical properties of Al predicted by the potentials in comparison with DFT data from the literature. There was no direct fit to any of these properties, although atomic configurations most relevant to some of the properties were represented in the training dataset. While both potentials agree with the DFT data well, the PINN potential tends to be more accurate for most properties. For the [110] self-interstitial dumbbell, the NN potential predicts an unstable configuration that spontaneously rotates to the [100] orientation, whereas the PINN potential correctly predicts such configurations to be metastable. Figure 3 shows the linear thermal expansion factor as a function of temperature predicted by the potentials in comparison with experimental data. The PINN potential displays good agreement with experiment without direct fit, whereas the NN potential overestimates the thermal expansion at high temperatures. (The discrepancies at low temperatures are due to the quantum effects that are not captured by classical simulations.) As another test, the radial distribution function and the bond angle distribution in liquid Al were computed at several temperatures for which experimental and/or DFT data are available (Supplementary Figs 4 and 5). In this case, both potentials were found to perform equally well. Any small deviations from the published DFT calculations are within the uncertainty of the different DFT flavors (exchange-correlation functionals).

**Table 2:** Aluminum properties predicted by the PINN and NN potentials

Property	DFT	NN	PINN
*E*_0_ (eV per atom)	−3.7480^a^	−3.3606	−3.3609
*a*_0_ (Å)	4.039^a,d^; 3.9725–4.0676^c^	4.0409	4.0396
*B* (GPa)	83^a^; 81^f^	80	79
*c*_11_ (GPa)	104^a^; 103–106^d^	108	117
*c*_12_ (GPa)	73^a^; 57–66^d^	66	60
*c*_44_ (GPa)	32^a^; 28–33^d^	25	32
*γ*_s_(100) (J m^−2^)	0.92^b^	0.897	0.899
*γ*_s_(110) (J m^−2^)	0.98^b^	0.986	0.952
*γ*_s_(111) (J m^−2^)	0.80^b^	0.837	0.819
$E_v^f$ (eV)	0.665–1.346^c^; 0.7^e^	0.640	0.678
$E_v^f$ (eV) unrelaxed	0.78^e^	0.71	0.77
$E_v^m$ (eV)	0.304−0.621^c^	0.627	0.495
$E_I^f$ (*T*_d_) (eV)	2.200–3.294^c^	2.683	2.840
$E_I^f$ (*O*_h_) (eV)	2.531–2.948^c^	1.600	2.367
$E_I^f$ 〈100〉 (eV)	2.295–2.607^c^	1.529	2.246
$E_I^f$ 〈110〉 (eV)	2.543–2.981^c^	1.529*	2.713
$E_I^f$ 〈111〉 (eV)	2.679–3.182^c^	2.631	2.815
*γ*_SF_ (mJ m^−2^)	134^i^; 146^g^; 158^h^	128	121
*γ*_us_ (mJ m^−2^)	162^j^; 169^i^; 175^h^	143	132

The potential predictions are compared with DFT calculations from the literature

*E*_0_ equilibrium cohesive energy, *a*_0_ equilibrium lattice parameter, *B* bulk modulus, *c*_*ij*_ elastic constants, *γ*_s_ surface energy, $E_v^f$ vacancy formation energy, $E_v^m$ vacancy migration barrier, $E_I^f$ interstitial formation energy for the tetrahedral (*T*_d_) and octahedral (*O*_h_) positions and split dumbbell configurations with different orientations, *γ*_SF_ intrinsic stacking fault energy, *γ*_us_ unstable stacking fault energy. All defect energies are statically relaxed unless otherwise indicated

^a^Ref. ^61^; ^b^ref.  ^62^; ^c^ref.  ^63^; ^d^ref.  ^64^; ^e^ref. ^65^; ^f^ref. ^66^ ; ^g^ref.  ^67^; ^h^ref.  ^68^; ^i^ref.  ^69^; ^j^ref. ^70^

*Unstable and flips to the 〈100〉 dumbbell orientation

**Fig. 4:**
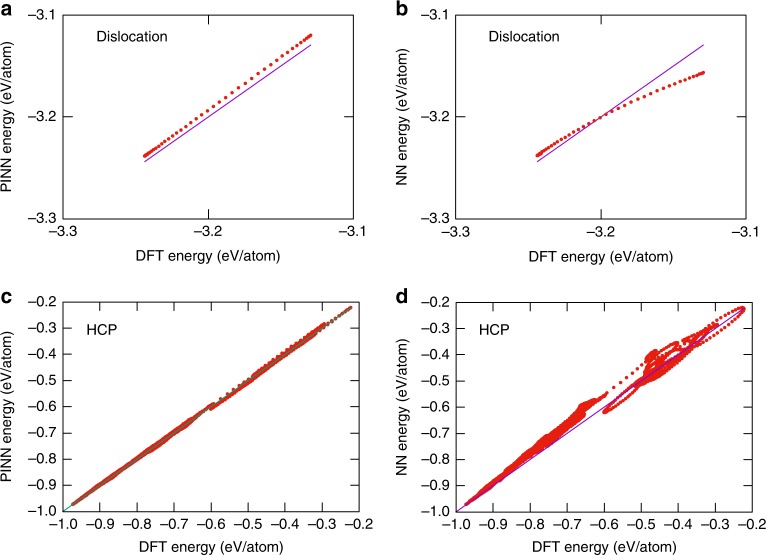
Testing of the NN and PINN potentials. **a**, **b** Energy of an edge dislocation in Al in NVE MD simulations starting at 700 K. **c**, **d** Energy of HCP Al in NVT MD simulations at 1000, 1500, 2000, and 4000 K. The energies predicted by the PINN (**a**, **c**) and NN (**b**, **d**) potentials are compared with DFT calculations from^20,21^. The straight lines represent the perfect fit

For testing purposes, we computed the energies of the remaining groups of structures that were part of the original DFT database^20,21^ but were not used here for training or validation. The full information about the testing dataset (26,425 supercells containing a total of 2,376,388 atoms) can be found in the Supplementary Table 3. For example, Fig. 4 compares the energies predicted by the potentials with DFT energies from high-temperature MD simulations for a supercell containing an edge dislocation or HCP Al. In both cases, the PINN potential is obviously more accurate. The remaining testing cases are presented in the Supplementary Figs. 6–10. Although there are cases where both potentials perform equally well, in most cases the PINN potential predicts the energies of unknown atomic configurations more accurately than the NN potential.

**Fig. 5:**
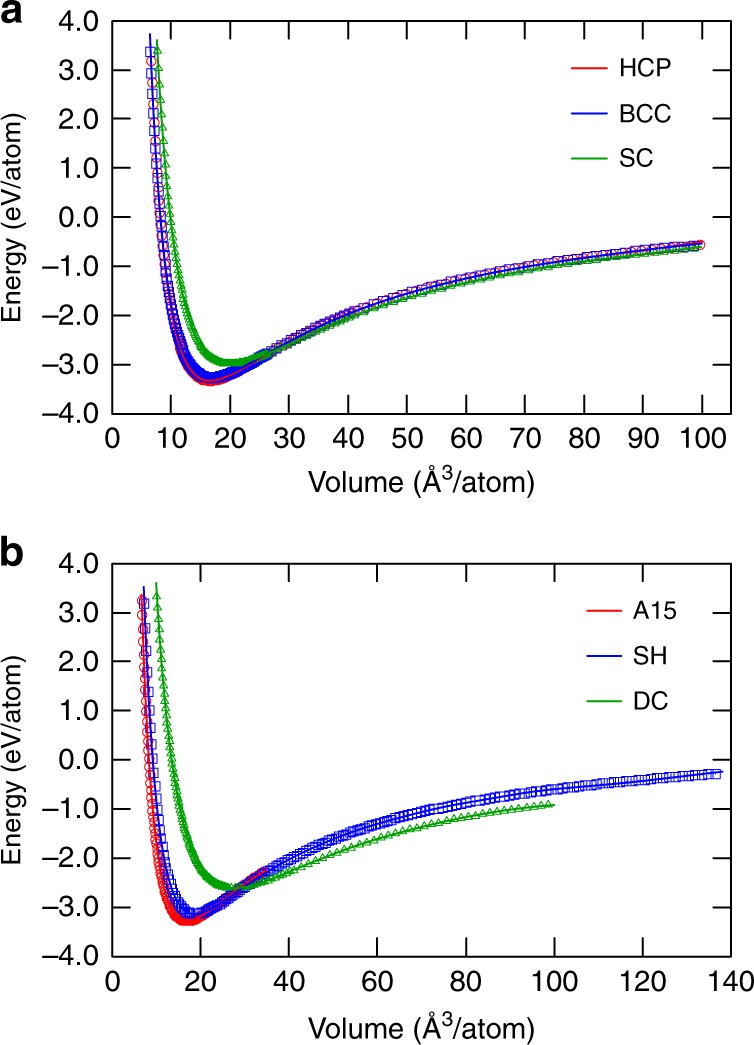
Energy–volume relations for Al crystal structures. Comparison of the energies predicted by the PINN potential (lines) and by DFT calculations (points). **a** Hexagonal close-packed (HCP), body-centered cubic (BCC), and simple cubic (SC) structures. **b** A15 (Cr_3_Si prototype), simple hexagonal (SH), and diamond cubic (DC) structures

**Fig. 6:**
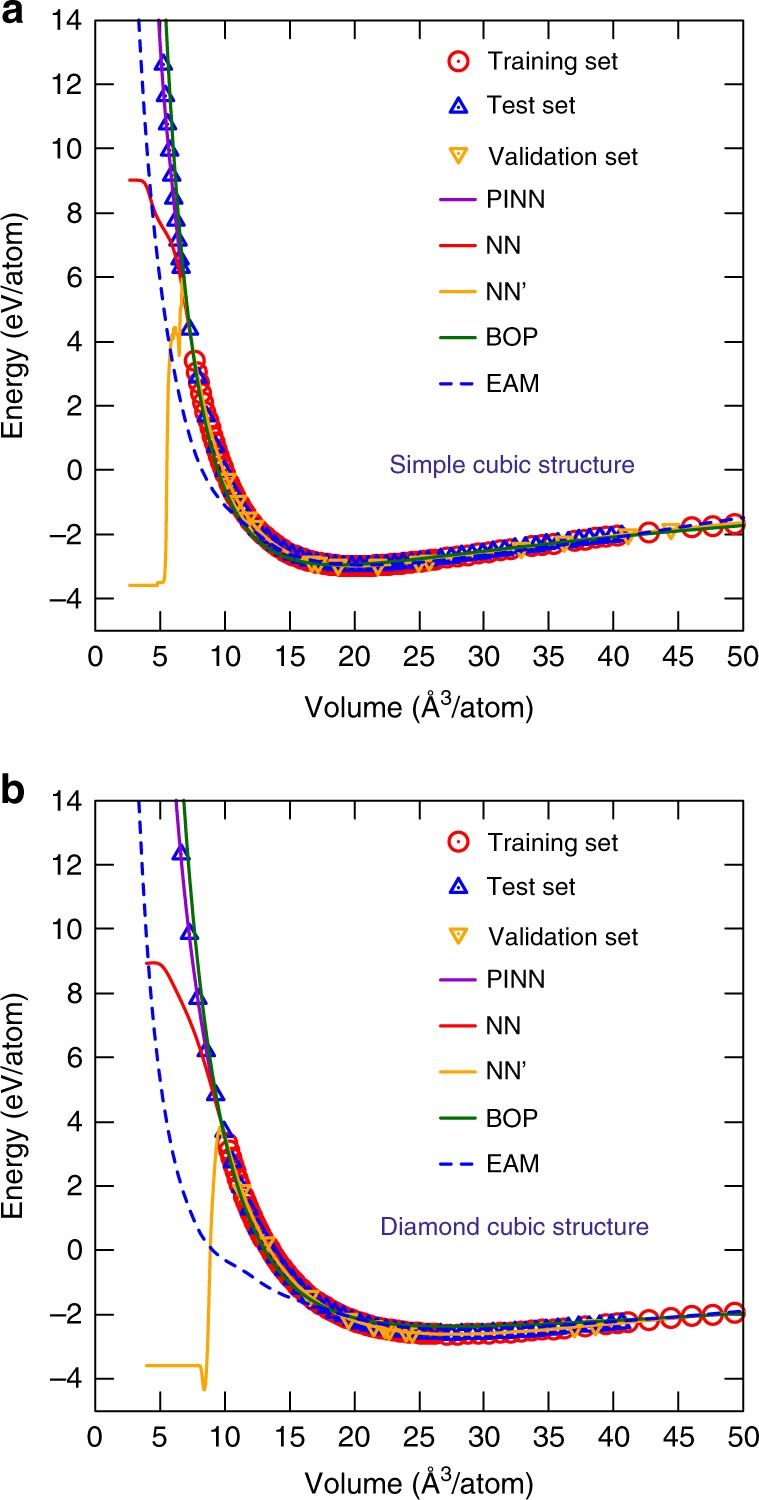
Zoom into the repulsive part of the energy–volume relations predicted by the PINN, NN, NN′, EAM, and BOP potentials (curves) and DFT calculations (points)

For further testing, the energies of the crystal structures of Al were computed for atomic volumes both within and beyond the training interval. Both potentials accurately reproduce the DFT energy–volume relations for all volumes spanned by the DFT database (Fig. 5 and Supplementary Fig. 3). However, extrapolation to larger or smaller volumes reveals significant differences. For example, the PINN potential correctly predicts that the crystal energy continues to rapidly increase under strong compression (repulsive interaction mode). In fact, the extrapolated PINN energy goes exactly through the new DFT points that were not included in the training or validation datasets, see examples in Fig. 6. By contrast, the energy predicted by the NN model immediately develops wiggles and strongly deviates from the physically meaningful repulsive behavior. Such artifacts were found for other structures as well.

To demonstrate that the unphysical behavior exhibited by the NN potential is not a specific feature of our structural parameters $G_i^l$ or the training method, we constructed another NN potential using a third-party NN-training package PROPhet^53^. This potential, which we refer to as NN′, uses the Behler-Parrinello symmetry functions^13^, which are different from our structural descriptor $G_i^l$. The NN-training algorithm is also different. A 47 × 18 × 18 × 1 network containing 1225 fitting parameters was trained on exactly the same DFT database to about the same accuracy as the NN and PINN potentials (Table 1). Figure 6 shows that the NN′ potential behaves in a similar manner as our NN potential, closely following the DFT energies within the training/validation domain and becoming unphysical as soon as we step outside this domain.

**Fig. 7:**
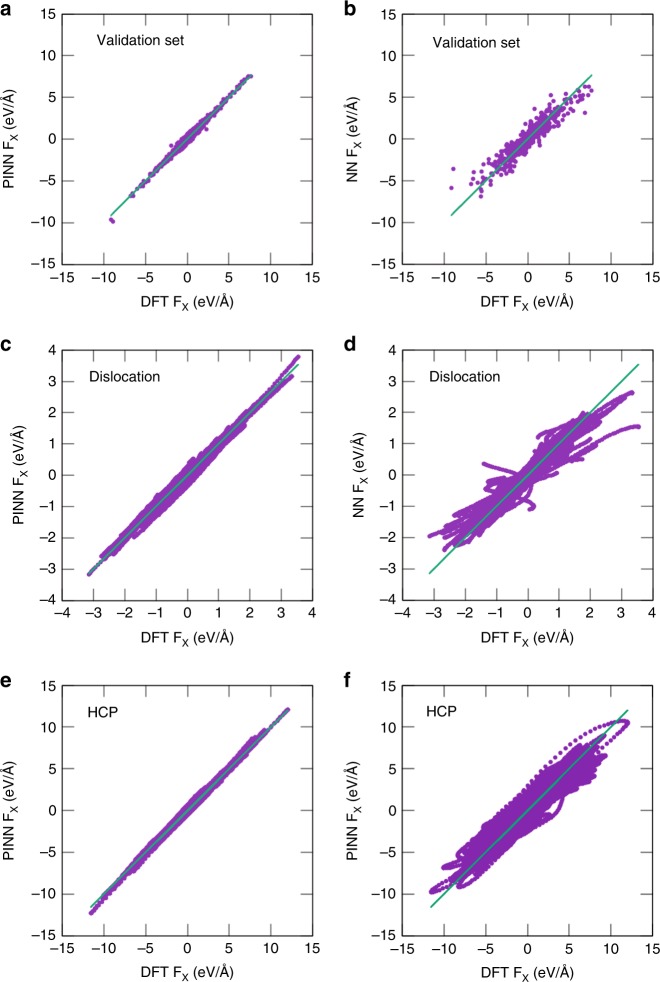
Testing of atomic force predictions. The *x*-component of atomic forces for **a**, **b** validation database, **c**, **d** edge dislocation in NVE MD simulations starting at 700 K, and **e**, **f** HCP Al in NVT MD simulations at 300, 600, 1000, 1500, 2000, and 4000 K. The forces predicted by the PINN (**a**, **c**, **e**) and NN (**b**, **d**, **f**) potentials are compared with DFT calculations from refs. ^20,21^. The straight lines represent the perfect fit. See Supplementary Figs 11–13 for all components of the forces

While the atomic forces were not used for either training or validation, they were compared with the DFT forces once the training was complete. For the validation dataset, this comparison probes the accuracy of interpolation, whereas for the testing dataset the accuracy of extrapolation. As expected, for the validation dataset the PINN forces are in better agreement with DFT calculations than the NN forces (RMSE ≈ 0.1 eV Å^−1^ versus ≈0.2 eV Å^−1^) as illustrated in Fig. 7a, b. For the testing dataset, the advantage of the PINN model in force predictions is even more significant. For example, for the dislocation and HCP cases discussed above, the PINN potential provides more accurate predictions (RMSE ≈ 0.1 eV Å^−1^) than the NN potential (RMSE ≈ 0.4 eV Å^−1^ for the dislocation and 0.6 eV Å^−1^ for the HCP case) (Fig. 7c, f). This advantage persists for all other groups of structures from the testing database.

**Fig. 8:**
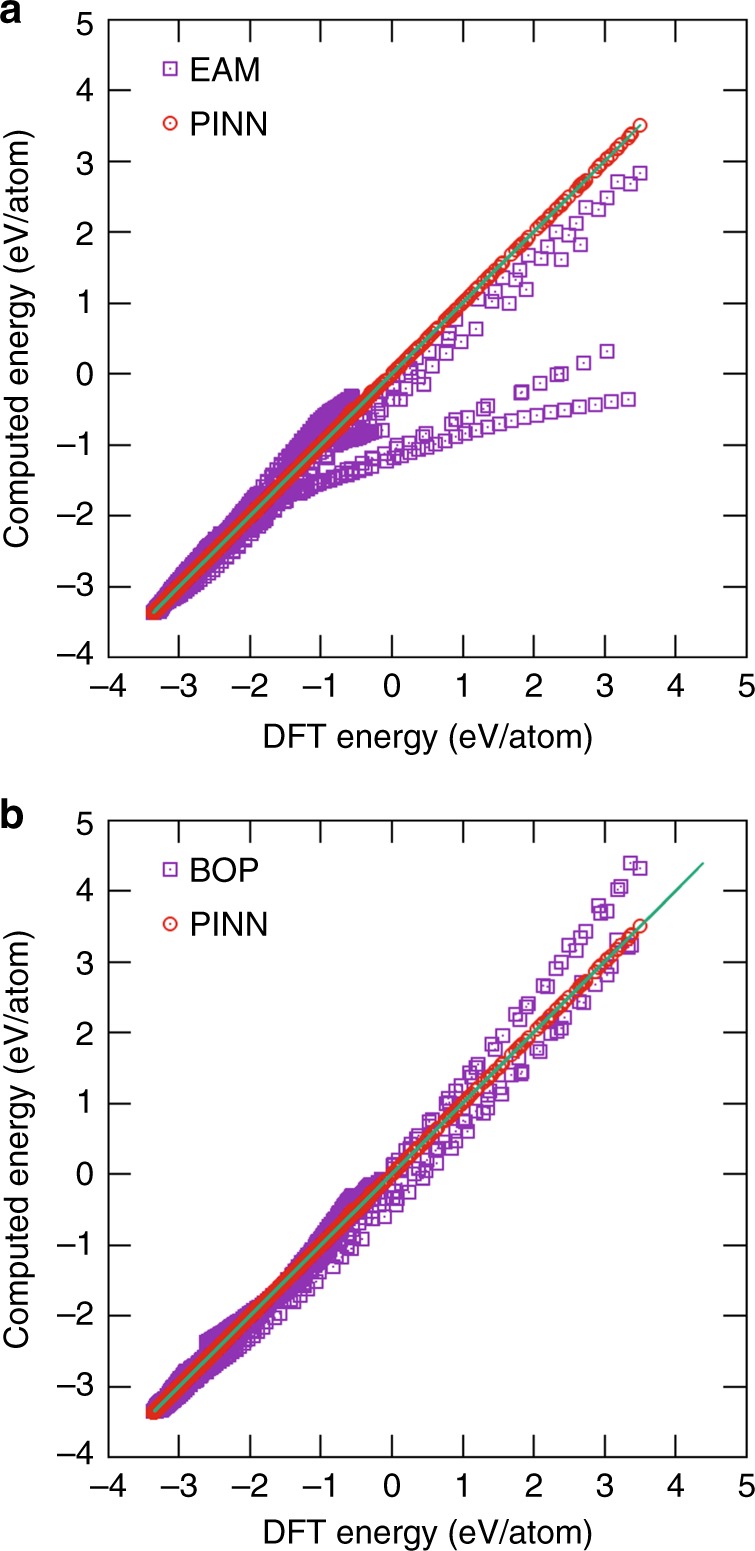
Comparison of DFT versus potentials. Energies of atomic configurations in the DFT database used for training and validation are compared with predictions of the **a** EAM Al potential^54^ and **b** BOP potential. The BOP parameters were fitted to the DFT database and permanently fixed. The PINN potential predictions are included for comparison. The straight line represents the perfect fit

It was also interesting to compare the PINN potential with traditional, parameter-based potentials for Al. One of them was the widely accepted EAM Al potential^54^ that had been fitted to a mix of experimental and DFT data. The other was a BOP potential of the same functional form as in the PINN model. Its parameters were fitted in this work using the same DFT database as for the PINN/NN potentials and then fixed once and for all. Figure 8 compares the DFT energies with the energies predicted by the EAM and BOP models across the entire set of reference configurations. The PINN predictions are shown for comparison. The plots demonstrate that the traditional, fixed-parameter models generally follow the correct trend but become increasingly less accurate as the structures deviate from the equilibrium, low-energy atomic configurations. The adaptivity to the local atomic environments built into the PINN potential greatly improves the accuracy.

## Discussion

The proposed PINN potential model is capable of achieving the same high accuracy in interpolating between DFT energies on the PES as the currently existing mathematical NN potentials. The construction of PINN potentials requires the same type of DFT database, is equally straightforward, and does not heavily rely on human intuition. However, extrapolation outside the domain of atomic configurations represented in the training database is now based on a physical model of interatomic bonding. As a result, the extrapolation becomes more reliable, or at least more failure-proof, than the purely mathematical extrapolation. The accuracy of interpolation can also be improved for the same reason. As an example, the PINN Al potential constructed in this paper demonstrates better accuracy of interpolation and significantly improved transferability than a regular NN potential with about the same number of parameters. The advantage of the PINN potential is especially strong for atomic forces, which are important for molecular dynamics. The potential could be used for accurate simulations of mechanical behavior and other processes in Al. Construction of general-purpose PINN potentials for Si and Ge is currently in progress.

We believe that the development of physics-based ML potentials is the best way forward in this field. Such potentials need not be limited to NNs or the particular BOP model adopted in this paper. Other regression methods can be employed and the interatomic bonding model can be made more sophisticated, or the other way round, simpler in the interest of speed.

Other modifications are envisioned in the future. For example, not all potential parameters are equally sensitive to local environments. To improve the computational efficiency, the parameters can be divided into two subsets^40^: local parameters **a**_*i*_ = (*a*_*i*1_, ..., *a*_*iλ*_) adjustable according to the local environments as discussed above, and global parameters **b** = (*b*_1_, ..., *b*_*μ*_) that are fixed after the optimization and used for all environments (as in the traditional potentials). The potential format now becomes

2 $$E_i = E_i\left( {{\mathbf{r}}_{i1},...,{\mathbf{r}}_{in},{\mathbf{a}}_i\left( {G_i^l({\mathbf{r}}_{i1},...,{\mathbf{r}}_{in})} \right),{\mathbf{b}}} \right).$$

During the training process, the global parameters **b** and the network weights and biases are optimized simultaneously, as shown in Fig. 1d. Extension of PINN potentials to binary and multicomponent systems is another major task for the future.

All ML potentials are orders of magnitude faster than straight DFT calculations but inevitably much slower than the traditional potentials. Preliminary tests indicate that PINN potentials are about 25% slower than the regular NN potentials for the same number of parameters, the extra overhead being due to the BOP calculation. However, the computational efficiency depends on the parallelization method and computer architecture. All computations reported in this paper utilized in-house software parallelized with MPI for training and with OpenMP for MD and MC simulations (see example in Supplementary Fig. 14). Collaborative work is underway to develop highly scalable HPC software packages for physically informed ML potential training and MD/MC simulations using multiple CPUs or GPUs, or both. The results will be reported in a forthcoming paper.

## Methods

### Local structural parameters

There are many possible ways of choosing local structural parameters^13–18,34,36^. After trying several options, the following set of $G_i^l$’s was selected. For an atom *i*, we define

3 $$g_i^{(m)} = \mathop {\sum}\limits_{j,k} {P_m} \left( {{\mathrm{cos}}\,\theta _{ijk}} \right)f(r_{ij})f(r_{ik}),m = 0,1,2,...,$$

where *r*_*ij*_ and *r*_*ik*_ are distances to atoms *j* and *k*, respectively, and *θ*_*ijk*_ is the angle between the bonds *ij* and *ik*. In Eq. (3), *P*_*m*_(*x*) is the Legendre polynomial of order *m* and

4 $$f(r) = \frac{1}{{\sigma ^3}}e^{ - (r - r_0)^2/\sigma ^2}f_c(r)$$

is a truncated Gaussian of width *σ* centered at point *r*_0_. The truncation function *f*_*c*_(*r*) is defined by

5 $$f_c(r) = \left\{ {\begin{array}{*{20}{l}} {\frac{{(r - r_c)^4}}{{d^4 + (r - r_c)^4}}} \hfill & {r \le r_c} \hfill \\ {0,} \hfill & {r \ge r_c.} \hfill \end{array}} \right.$$

This function and its derivatives up to the third go to zero at a cutoff distance *r*_*c*_. The parameter *d* controls the truncation range.

For example, *P*_0_(*x*) = 1 and $g_i^{(0)}$ characterizes the local atomic density near atom *i*. Likewise, *P*_1_(*x*) = *x* and $g_i^{(1)}$ can be interpreted as the dipole moment of a set of unit charges placed at the atomic positions *j* and *k*. As such, this parameter measures the degree of local deviation from spherical symmetry in the environment ($g_i^{(1)} = 0$ for spherical symmetry). For *m* = 2, we have *P*_2_(*x*) = (3*x*^2^ − 1)/2 and $g_i^{(2)}$ is related to the quadrupole moment of a set of unit charges placed at the atomic positions around atom *i*. We found that polynomials up to degree *m* = 6 should be included to accurately represent the diverse atomic environment. Each $g_i^{(l)}$ is computed for several values of *σ* and *r*_0_ spanning a range of interatomic distances. For each atom, the set of *k* $g_i^{(m)}$’s obtained is arranged in a one-dimensional array $(G_i^1,G_i^2,...,G_i^k)$. In this work we chose *σ* = 1.0 and used polynomials with *m* = 0, 1, 2, 4, 6 for 12 *r*_0_ values, giving a total of *k* = 60 $G_i^l$’s.

### The BOP potential

In the BOP model adopted in this work, the energy of an atom *i* is postulated in the form

6 $$E_i = \frac{1}{2}\mathop {\sum}\limits_{j \ne i} {\left[ {e^{A_i - \alpha _ir_{ij}} - S_{ij}b_{ij}e^{B_i - \beta _ir_{ij}}} \right]} f_c(r_{ij}) + E_i^{(p)},$$

where *r*_*ij*_ is the distance between atoms *i* and *j* and the summation is over all atom *j* other than *i* within the cutoff radius *r*_*c*_. The bond-order parameter *b*_*ij*_ is taken in the form

7 $$b_{ij} = (1 + z_{ij})^{ - 1/2},$$

where

8 $$z_{ij} = a_i^2\mathop {\sum}\limits_{k \ne i,j} {S_{ik}} ({\mathrm{cos}}\theta _{ijk} + h_i)^2f_c(r_{ik})$$

represents the number of chemical bonds (other than *ij*) formed by atom *i*. Larger *z*_*ij*_ values (more bonds) lead to a smaller *b*_*ij*_ and thus weaker *ij* bond.

The screening factor *S*_*ij*_ reduces the strength of bonds by surrounding atoms. For example, when counting the bonds in Eq. (8), we screen them by *S*_*ik*_, so that strongly screened bonds contribute less to *z*_*ij*_. The screening factor *S*_*ij*_ is given by

9 $$S_{ij} = \mathop {\prod}\limits_{k \ne i,j} S_{ijk},$$

where the partial screening factor *S*_*ijk*_ represents the contribution of a neighboring atom *k* (different from *i* and *j*) to the screening of the bond *ij*. *S*_*ijk*_ is given by

10 $$S_{ijk} = 1 - f_c(r_{ik} + r_{jk} - r_{ij})e^{ - \lambda _i^2(r_{ik} + r_{jk} - r_{ij})}.$$

It has the same value for all atoms *k* located on the surface of an imaginary spheroid whose poles coincide with the atoms *i* and *j*. For all atoms *k* outside this cutoff spheroid, on which *r*_*ik*_ + *r*_*jk*_ − *r*_*ij*_ = *r*_*c*_, we have *S*_*ijk*_ = 1 — such atoms are too far away to screen the bond. If an atom *k* is placed on the line between the atoms *i* and *j*, we have *r*_*ik*_ + *r*_*jk*_ − *r*_*ij*_ = 0 and *S*_*ijk*_ is small — the bond *ij* is strongly screened (almost broken) by the atom *k*. This behavior reasonably reflects the nature of chemical bonding.

Finally, the promotion energy $E_i^{(p)}$ is taken in the form

11 $$E_i^{(p)} = - \sigma _i\left( {\mathop {\sum}\limits_{j \ne i} {S_{ij}} b_{ij}f_c(r_{ij})} \right)^{1/2}.$$

For a covalent material, $E_i^{(p)}$ accounts for the energy cost of changing the electronic structure of a free atoms before it forms chemical bonds. For example, for group IV elements, this is the cost of the s^2^p^2^ → sp^3^ hybridization. On the other hand, $E_i^{(p)}$ can be interpreted as the embedding energy

12 $$F(\bar \rho _i) = - \sigma _i\left( {\bar \rho _i} \right)^{1/2}$$

appearing in the EAM formalism^1,2^. Here, the host electron density on atom *i* is given by $\bar \rho _i = \mathop {\sum}\nolimits_{j \ne i} {S_{ij}} b_{ij}\, f_c(r_{ij})$. Due to this feature, this BOP model can be applied to both covalent and metallic systems.

The BOP functions depend on eight parameters *A*_*i*_, *B*_*i*_, *α*_*i*_, *β*_*i*_, *a*_*i*_, *h*_*i*_, *σ*_*i*_, and *λ*_*i*_, which constitute the parameter set (*p*_1_, ..., *p*_*m*_) with *m* = 8. The cutoff parameters were fixed at *r*_*c*_ = 6 Å and *d* = 1.5 Å.

### The neural network and training procedures

The feedforward NN contained two hidden layers and had the 60 × 15 × 15 × 8 architecture for the PINN potential and 60 × 16 × 16 × 1 for the NN potential. The number of nodes in the hidden layers was chosen to reach the target accuracy of about 3-4 meV/atom without overfitting.

The training/validation database consisted of DFT total energies for a set of supercells. The DFT calculations were performed using projector-augmented wave (PAW) pseudopotentials as implemented in the electronic structure Vienna Ab initio Simulation Package (VASP)^55,56^. The generalized gradient approximation (GGA) was used in conjunction with the Perdew, Burke, and Ernzerhof (PBE) density functional^57,58^. The plane-wave basis functions up to a kinetic energy cutoff of 520 eV were used, with the *k*-point density chosen to achieve convergence to a few meV per atom level. Further details of the DFT calculations can be found in refs. ^20,21^. The energy of a given supercell *s*, $E^s = \mathop {\sum}\nolimits_i {E_i^s}$, predicted by the potential was compared with the DFT energy $E_{{\mathrm{DFT}}}^s$. Note that the original $E_{{\mathrm{DFT}}}^s$ values were not corrected to remove the energy of a free atom. To facilitate comparison with literature data, prior to the training all DFT energies were uniformly shifted by 0.38446 eV per atom to match the experimental cohesive energy of Al, 3.36 eV per atom^59^. The NN was trained by adjusting its weights *w*_*εκ*_ and biases *b*_*κ*_ to minimize the objective function

13 $${\cal{E}} = \mathop {\sum}\limits_s {\left( {E^s - E_{{\mathrm{DFT}}}^s} \right)^2} + \tau \left( {\mathop {\sum}\limits_{\epsilon \kappa } {\left| {w_{\epsilon \kappa }} \right|^2} + \mathop {\sum}\limits_\kappa {\left| {b_\kappa } \right|^2} } \right) + \gamma \left( {\mathop {\sum}\limits_\eta {\left| {p_\eta - \bar p_\eta } \right|^2} } \right).$$

The second term was added to avoid overfitting by controlling the magnitudes of the weights and biases. The parameter *τ* controls the degree of regularization. The third term ensures that the variations of the PINN parameters relative to their database-averaged values $\bar p_\eta$ remain small. The minimization of ${\cal{E}}$ was implemented by the Davidson–Fletcher–Powell algorithm of unconstrained optimization. The optimization was repeated several times starting from different random states and the solution with the smallest ${\cal{E}}$ was selected as final. The PINN and NN forces were computed by the finite-difference method.

## Supplementary Information

Supplementary Information

Peer Review File

## Data Availability

All data that support the findings of this study are available in the Supplementary Information file or from the corresponding author upon reasonable request.

## References

[1] Daw MS, Baskes MI (1984). Embedded-atom method: derivation and application to impurities, surfaces, and other defects in metals. Phys. Rev. B.

[2] Daw MS, Baskes MI (1983). Semiempirical, quantum mechanical calculation of hydrogen embrittlement in metals. Phys. Rev. Lett..

[3] Mishin, Y. in *Handbook of Materials Modeling* (ed. Yip, S.), Ch. 2.2, 459–478 (Springer, Dordrecht, 2005).

[4] Baskes MI (1987). Application of the embedded-atom method to covalent materials: a semi-empirical potential for silicon. Phys. Rev. Lett..

[5] Mishin Y, Mehl MJ, Papaconstantopoulos DA (2005). Phase stability in the Fe-Ni system: investigation by first-principles calculations and atomistic simulations. Acta Mater..

[6] Liang T, Devine B, Phillpot SR, Sinnott SB (2012). Variable charge reactive potential for hydrocarbons to simulate organic-copper interactions. J. Phys. Chem..

[7] Brenner DW (1990). Empirical potential for hyrdocarbons for use in simulating the chemical vapor deposition of diamond films. Phys. Rev. B.

[8] Brenner DW (2000). The art and science of an analytical potential. Phys. Stat. Solidi (b).

[9] Stuart SJ, Tutein AB, Harrison JA (2000). A reactive potential for hydrocarbons with intermolecular interactions. J. Chem. Phys..

[10] van Duin ACT, Dasgupta S, Lorant F, Goddard WA (2001). Reaxff: a reactive force field for hydrocarbons. J. Phys. Chem..

[11] Mishin Y, Asta M, Li J (2010). Atomistic modeling of interfaces and their impact on microstructure and properties. Acta Mater..

[12] Mueller, T., Kusne, A. G. & Ramprasad, R. in *Reviews in Computational Chemistry* (eds Parrill, A. L. & Lipkowitz, K. B.), Vol. 29, Ch. 4, 186–273 (Wiley, 2016).

[13] Behler J, Parrinello M (2007). Generalized neural-network representation of high-dimensional potential-energy surfaces. Phys. Rev. Lett..

[14] Behler J, Martonak R, Donadio D, Parrinello M (2008). Metadynamics simulations of the high-pressure phases of silicon employing a high-dimensional neural network potential. Phys. Rev. Lett..

[15] Behler J (2011). Neural network potential-energy surfaces in chemistry: a tool for large-scale simulations. Phys. Chem. Chem. Phys..

[16] Behler J (2011). Atom-centered symmetry functions for constructing high-dimensional neural network potentials. J. Chem. Phys..

[17] Behler J (2015). Constructing high-dimensional neural network potentials: a tutorial review. Int. J. Quant. Chem..

[18] Behler J (2016). Perspective: machine learning potentials for atomistic simulations. Phys. Chem. Chem. Phys..

[19] Bartok A, Payne MC, Kondor R, Csanyi G (2010). Gaussian approximation potentials: the accuracy of quantum mechanics, without the electrons. Phys. Rev. Lett..

[20] Botu V, Ramprasad R (2015). Adaptive machine learning framework to accelerate ab initio molecular dynamics. Int. J. Quant. Chem..

[21] Botu V, Ramprasad R (2015). Learning scheme to predict atomic forces and accelerate materials simulations. Phys. Rev. B.

[22] Wood MA, Thompson AP (2018). Extending the accuracy of the SNAP interatomic potential form. J. Chem. Phys..

[23] Raff LM, Komanduri R, Hagan M, Bukkapatnam STS (2012). Neural Networks in Chemical Reaction Dynamics.

[24] Blank TB, Brown SD, Calhoun AW, Doren DJ (1995). Neural network models of potential energy surfaces. J. Chem. Phys..

[25] Payne, M., Csanyi, G. & de Vita, A. in *Handbook of Materials Modeling* (ed. Yip, S.), 2763–2770 (Springer, Dordrecht, 2005).

[26] Li Z, Kermode JR, De Vita A (2015). Molecular dynamics with on-the-fly machine learning of quantum-mechanical forces. Phys. Rev. Lett..

[27] Glielmo A, Sollich P, de Vita A (2017). Accurate interatomic force fields via machine learning with covariant kernels. Phys. Rev. B.

[28] Dawes R, Thompson DL, Wagner AF, Minkoff M (2008). Interpolating moving least-squares methods for fitting potential energy surfaces: a strategy for efficient automatic data point placement in high dimensions. J. Chem. Phys..

[29] Seko A, Takahashi A, Tanaka I (2015). First-principles interatomic potentials for ten elemental metals via compressed sensing. Phys. Rev. B.

[30] Mizukami W, Hebershon S, Tew DP (2015). A compact and accurate semi-global potential energy surface for malonaldehyde from constrained least squares regression. J. Chem. Phys..

[31] Chmiela S, Sauceda HE, Muller KR, Tkatchenko A (2018). Towards exact molecular dynamics simulations with machine-learned force fields. Nat. Commun..

[32] Bholoa A, Kenny SD, Smith R (2007). A new approach to potential fitting using neural networks. Nucl. Instrum. Methods Phys. Res..

[33] Sanville E, Bholoa A, Smith R, Kenny SD (2008). Silicon potentials investigated using density functional theory fitted neural networks. J. Phys. Condens. Matter.

[34] Eshet H, Khaliullin RZ, Kuhle TD, Behler J, Parrinello M (2010). Ab initio quality neural-network potential for sodium. Phys. Rev. B.

[35] Handley CM, Popelier PLA (2010). Potential energy surfaces fitted by artificial neural networks. J. Phys. Chem. A.

[36] Sosso GC, Miceli G, Caravati S, Behler J, Bernasconi M (2012). Neural network interatomic potential for the phase change material GeTe. Phys. Rev. B.

[37] Schutt, K. T., Sauceda, H. E., Kindermans, P. J., Tkatchenko, A. & Muller, K. R. Schnet—a deep learning architecture for molecules and materials. *J. Chem. Phys*. **148**, 241722 (2018). 10.1063/1.5019779 29960322

[38] Imbalzano G (2018). Automatic selection of atomic fingerprints and reference configurations for machine-learning potentials. J. Chem. Phys..

[39] Bartok AP, Kermore J, Bernstein N, Csanyi G (2018). Machine learning a general purpose interatomic potential for silicon. Phys. Rev. X.

[40] Malshe M (2008). Parametrization of analytic interatomic potential functions using neural networks. J. Chem. Phys..

[41] Tersoff J (1988). New empirical approach for the structure and energy of covalent systems. Phys. Rev. B.

[42] Tersoff J (1988). Empirical interatomic potential for silicon with improved elastic properties. Phys. Rev. B.

[43] Tersoff J (1989). Modeling solid-state chemistry: interatomic potentials for multicomponent systems. Phys. Rev. B.

[44] Bereau T, Andrienko D, von Lilienfeld OA (2015). Transferable atomic multipole machine learning models for small organic molecules. J. Chem. Theor. Comput..

[45] Bereau T, DiStasio RA, Tkatchenko A, von Lilienfeld OA (2018). Non-covalent interactions across organic and biological subsets of chemical space: physics-based potentials parametrized from machine learning. J. Chem. Phys..

[46] Kranz JJ, Kubillus M, Ramakrishnan R, von Lilienfeld OA (2018). Generalized density-functional tight-binding repulsive potentials from unsupervised machine learning. J. Chem. Theor. Comput..

[47] Glielmo A, Zeni C, de Vita A (2018). Efficient nonparametric *n*-body force fields from machine learning. Phys. Rev. B.

[48] Hornik K, Stinchcombe M, White H (1989). Multilayer feedforward networks are universal approximation. Neural Netw..

[49] Pinkus A (1999). Approximation theory of the MLP model in neural networks. Acta Numer..

[50] Oloriegbe, S. Y. *Hybrid Bond-Order Potential for Silicon*. Ph.D. thesis (Clemson University, Clemson, 2008).

[51] Gillespie BA (2007). Bond-order potential for silicon. Phys. Rev. B.

[52] Drautz R (2007). Analytic bond-order potentials for modelling the growth of semiconductor thin films. Prog. Mater. Sci..

[53] Kolb B, Lentz LC, Kolpak AM (2017). Discovering charge density functionals and structure-property relationships with PROPhet: a general framework for coupling machine learning and first-principles methods. Sci. Rep..

[54] Mishin Y, Farkas D, Mehl MJ, Papaconstantopoulos DA (1999). Interatomic potentials for monoatomic metals from experimental data and ab initio calculations. Phys. Rev. B.

[55] Kresse G, Furthmüller J (1996). Efficiency of ab-initio total energy calculations for metals and semiconductors using a plane-wave basis set. Comput. Mat. Sci..

[56] Kresse G, Joubert D (1999). From ultrasoft pseudopotentials to the projector augmented-wave method. Phys. Rev. B.

[57] Perdew JP (1992). Atoms, molecules, solids, and surfaces: applications of the generalized gradient approximation for exchange and correlation. Phys. Rev. B.

[58] Perdew JP, Burke K, Ernzerhof M (1996). Generalized gradient approximation made simple. Phys. Rev. Lett..

[59] Kittel C (1986). Introduction to Sold State Physics.

[60] Touloukian, Y. S., Kirby, R. K., Taylor, R. E. & Desai, P. D. (eds.) *Thermal Expansion: Metallic Elements and Alloys*, Vol. 12 (Plenum, New York, 1975).

[61] de Jong M (2015). Charting the complete elastic properties of inorganic crystalline compounds. Sci. Data.

[62] Tran R (2016). Surface energies of elemental crystals. Sci. Data.

[63] Qiu R (2017). Energetics of intrinsic point defects in aluminium via orbital-free density functional theory. Philos. Mag..

[64] Zhuang H, Chen M, Carter EA (2016). Elastic and thermodynamic properties of complex Mg-Al intermetallic compounds via orbital-free density functional theory. Phys. Rev. Appl..

[65] Iyer M, Gavini V, Pollock TM (2014). Energetics and nucleation of point defects in aluminum under extreme tensile hydrostatic stresses. Phys. Rev. B.

[66] Sjostrom T, Crockett S, Rudin S (2016). Multiphase aluminum equations of state via density functional theory. Phys. Rev. B.

[67] Devlin JF (1974). Stacking fault energies of Be, Mg, Al, Cu, Ag, and Au. J. Phys. F: Met. Phys..

[68] Ogata S, Li J, Yip S (2002). Ideal pure shear strength of aluminum and copper. Science.

[69] Jahnatek M, Hafner J, Krajci M (2009). Shear deformation, ideal strength, and stacking fault formation of fcc metals: a density-functional study of Al and Cu. Phys. Rev. B.

[70] Kibey S, Liu JB, Johnson DD, Sehitoglu H (2007). Predicting twinning stress in fcc metals: linking twin-energy pathways to twin nucleation. Acta Mater..

