# Supplementary material for: Physically informed artificial neural networks for atomistic modeling of materials
Source: Nat Commun. 2019 May 28;10:2339. doi: 10.1038/s41467-019-10343-5 (PMC6538760; doi:10.1038/s41467-019-10343-5)
Supplement: Supplementary file 1 — Supplementary Information [file 41467_2019_10343_MOESM1_ESM.pdf]

Supplementary Information

Physically-informed artificial neural networks for atomistic modeling of materials

G. P. Purja Pun et al.

Supplementary Table 1 Al DFT database used in this work. The DFT data indicated by an asterisk were computed in this work. The remaining data were randomly selected from the database generated by Botu *et al.* [1, 2]. The structures are divided into datasets and further into groups according to the structure type and physical conditions (temperature, deformation). For NVE simulations, the table indicates the temperature of initial thermalization with ideal atomic positions.

Dataset	Structure	Group	Physical condition	N_A	N_{tv}
Crystals	FCC*	25	Isotropic strain at 0 K	4	174
	BCC*	14	Isotropic strain at 0 K	2	174
	HCP*	34	Isotropic strain at 0 K	4	174
	SC*	38	Isotropic strain at 0 K	8	161
	DC*	23	Isotropic strain at 0 K	8	152
	FCC*	26	Uniaxial $\langle 100 \rangle$ at 0 K	4	81
	A15*	13	Isotropic strain at 0 K	8	137
	SH*	35	Isotropic strain at 0 K	1	169
	FCC*	27	Uniaxial $\langle 100 \rangle$ at 0 K	1	61
	FCC*	28	Uniaxial $\langle 111 \rangle$ at 0 K	24	60
FCC 1	FCC ($a = 4.036$ Å)	24	NVE-MD (2500 K)	32	60
	FCC ($a = 4.036$ Å)	24	NVE-MD (700 K)	32	60
	FCC ($a = 3.302$ Å)*	37	NVT-MD (4000 K)	32	60
	FCC ($a = 3.530$ Å)*	36	NVT-MD (4000 K)	32	60
FCC 2	FCC ($a = 3.75$ Å)	7	NVE-MD (1200 K)	32	60
	FCC ($a = 3.96$ Å)	8	NVE-MD(700 K)	32	60
	FCC ($a = 4.00$ Å)	12	NVE-MD(700 K)	32	60
	FCC ($a = 4.10$ Å)	10	NVE-MD(700 K)	32	60
	FCC ($a = 4.15$ Å)	9	NVE-MD(700 K)	32	60
	FCC ($a = 4.35$ Å)	11	NVE-MD(1200 K)	32	60
Surfaces	Surface (100)	1	NVE-MD (700 K)	144	50
	Surface (110)	2	NVE-MD (700 K)	128	60
	Surface (111)	3	NVE-MD (700 K)	16	60
	Surface (100)	4	NVE-MD (1000 K)	108	60
	Surface (311)	5	NVE-MD (1000 K)	88	60
	Surface (111)	6	NVE-MD (1000 K)	108	60
Defects	1 Vacancy	44	NVE-MD (700 K)	31	210
	1 adatom on (100)	40	NVE-MD (700 K)	76	60
	2 adatoms on (111)	41	NVE-MD (700 K)	66	60
	Dimer on (111)	42	NVE-MD (700,2000 K)	66	60
	Trimer on (111)	43	NVE-MD (700,2000 K)	103	60

Continued in Table 2

Supplementary Table 2 Aluminum DFT database (continued from Table 1).

Dataset	Structure	Group	Physical condition	N_A	N_{tv}
Clusters	Dimer	20	NVE-MD (300 K)	2	60
	2.5 Å cluster	15	NVE-MD (300 K)	6	60
	4 Å cluster	16	NVE-MD (300 K)	13	60
	4.5 Å cluster	18	NVE-MD (300 K)	19	60
	5 Å cluster*	13	NVE-MD (1200 K)	42	60
	6.5 Å cluster*	19	NVE-MD (1200 K)	79	60
	Small icosahedron*	21	NVE-MD (900 K)	55	60
	Wulff cluster*	22	NVE-MD (1000 K)	79	60
	Wulff cluster*	22	NVE-MD (2000 K)	79	60
Interfaces	GB (510)	23	NVE-MD (700 K)	70	60
	GB (111)	19	NVE-MD (700 K)	24	60
	GB (210)	20	NVE-MD (700 K)	60	60
	GB (310)	21	NVE-MD (700 K)	42	60
	GB (320)	22	NVE-MD (700 K)	96	60
	SF⟨211⟩(111)*	1	Only atomic relaxation	30	60
Total					3649

N_A - number of atoms per supercell

N_{tv} - number of configurations for training and validation

Notations: BCC (body centered cubic), HCP (hexagonal closed packed)

SC (simple cubic), DC (diamond cubic), SH (simple hexagonal)

GB (grain boundary), SF (stacking fault). a is the cubic lattice parameter of the FCC structure

Supplementary Table 3 Al DFT database used for testing. The data was extracted from the database generated by Botu *et al.* [1, 2]. The structures are divided into datasets and further into groups according to the structure type and physical conditions (temperature, deformation). For NVE simulations, the table indicates the temperature of initial thermalization with ideal atomic positions.

Dataset	Structure	Run-type	N_A	N_t
BCC	BCC ($a = 2.621$ Å)	NVT-MD^a	54	2589
	BCC ($a = 2.802$ Å)	NVT-MD^a	54	2607
HCP	HCP† ($a = 1.847$ Å)	NVT-MD^a	32	3880
	HCP† ($a = 1.975$ Å)	NVT-MD^a	32	3853
FCC 3	FCC	NPT-MD (300,600,900 K)	32	6330
	FCC (EAM generated)	NPT-MD (300,600,900 K)	256	30
Defects	2 Vacancies	NVE-MD (700 K)	254	578
	6 Vacancies	NVE-MD (700 K)	860	165
	8 adatoms on (111)	NVE-MD (1500 K)	253	1420
	15 adatoms on (111)	NVE-MD (1500 K)	260	1397
	Dislocation	NVE-MD (700 K)	378	50
Clusters	8 Å cluster	NVE-MD (1200 K)	135	1707
	10 Å cluster	NVE-MD (1200 K)	249	249
	Octahedron cluster	NVE-MD (1000 K)	201	1570
Total				26425

N_A - number of atoms per supercell

N_t - number of configurations for testing

^{a} 300 K, 600 K, 1000 K, 1500 K, 2000 K and 4000 K

† $c/a = 1.648$

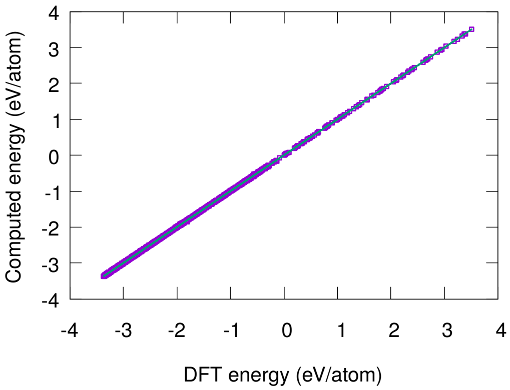

a

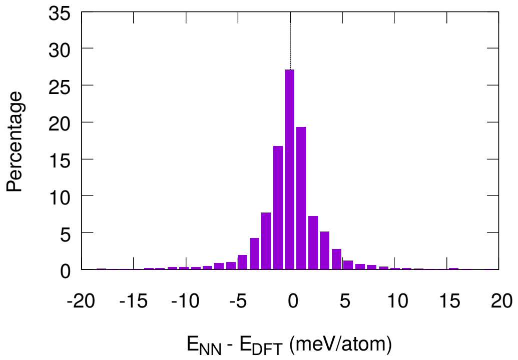

b

Supplementary Fig. 1 **a** Energies of atomic configurations in the training dataset computed with the mathematical NN potentials versus DFT energies. The straight line represents the perfect fit. **b** Error distribution in the training dataset.

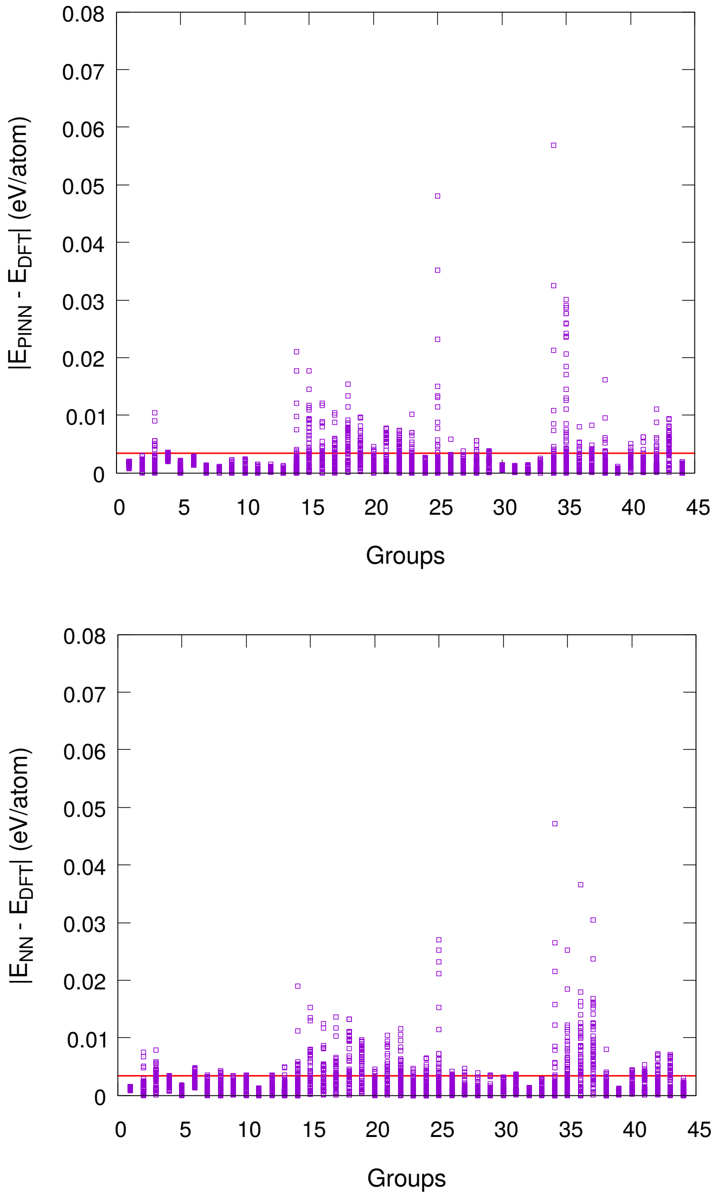

a **b**

Supplementary Fig. 2 Absolute deviations of energies predicted by the PINN (**a**) and NN (**b**) potentials from the DFT energies in individual groups of the training dataset. (Refer to Tables 1 and 2 for the group numbers). The red line marks the RMSE.

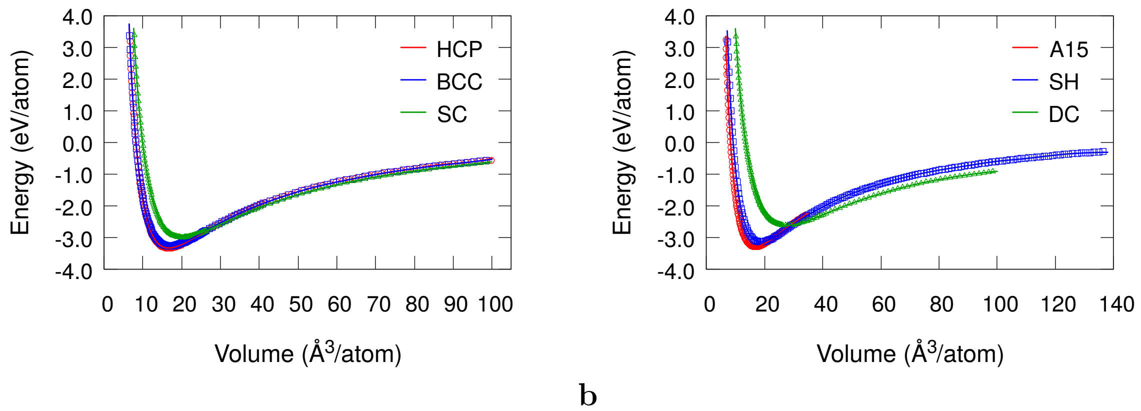

Supplementary Fig. 3 Energy-volume relations for Al crystal structures predicted by the NN potential (lines) and by DFT calculations (points). **a** Hexagonal close-packed (HCP), body-centered cubic (BCC), and simple cubic (SC) structures. **b** A15 (Cr_3Si prototype), simple hexagonal (SH), and diamond cubic (DC) structures.

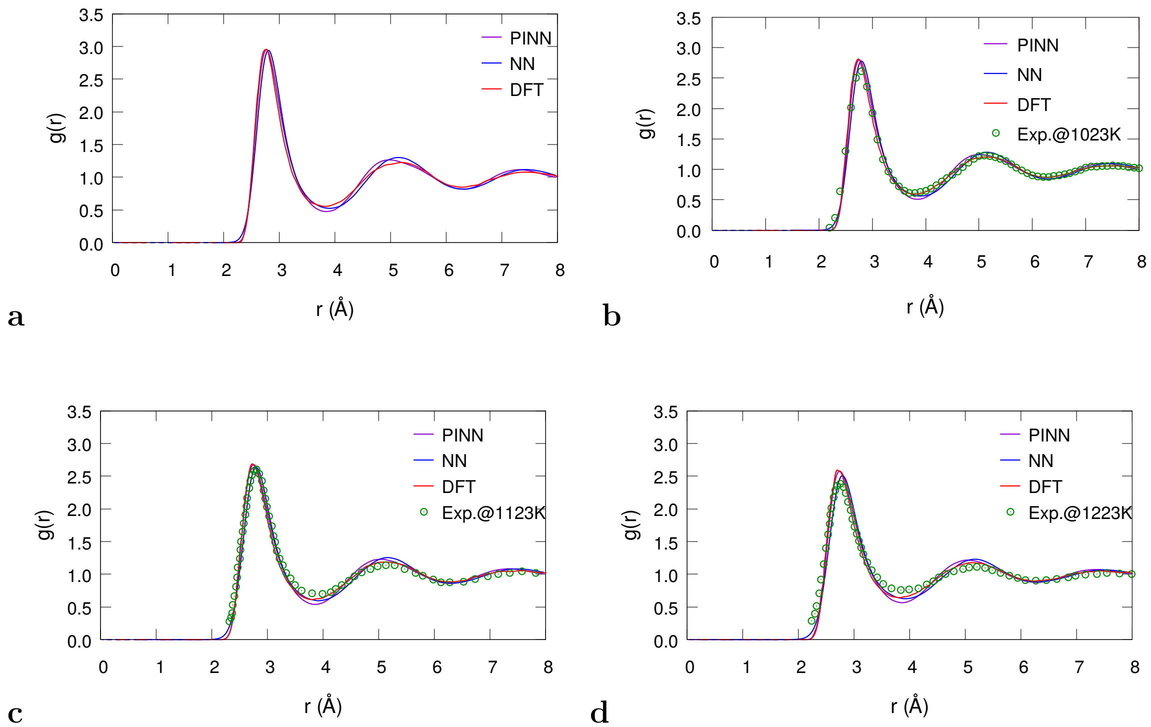

Supplementary Fig. 4 Radial distribution functions $g(r)$ in liquid Al at the temperatures of **a** 875 K, **b** 1000 K, **c** 1125 K and **d** 1250 K predicted by the PINN and NN potentials in comparison with experimental data [3] and DFT calculations (Ref. [4] and references therein).

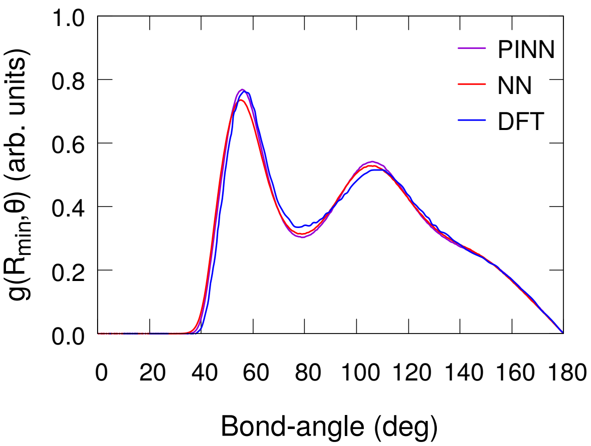

Supplementary Fig. 5 Bond angle distribution, $g(R_{min}, \theta)$, in liquid aluminum at 1000 K in comparison with DFT calculations [5]. The calculation included the neighbors within the first minimum R_{min} of the radial distribution function (cf. Supplementary Figure 4).

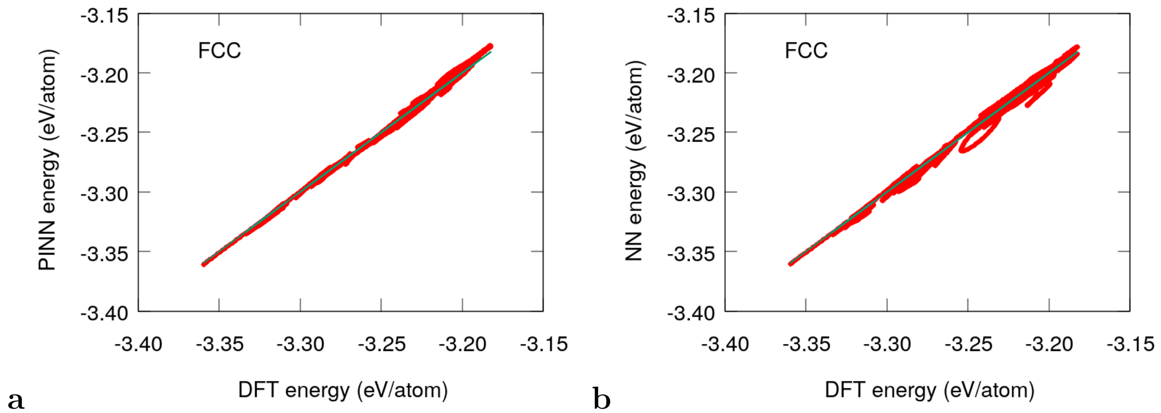

Supplementary Fig. 6 Energy of FCC Al in NPT MD simulations at the temperatures of 300 K and 600 K The energies predicted by the PINN (**a**) and NN (**b**) potentials are compared with DFT calculations from [1, 2]. The straight lines represent the perfect fit.

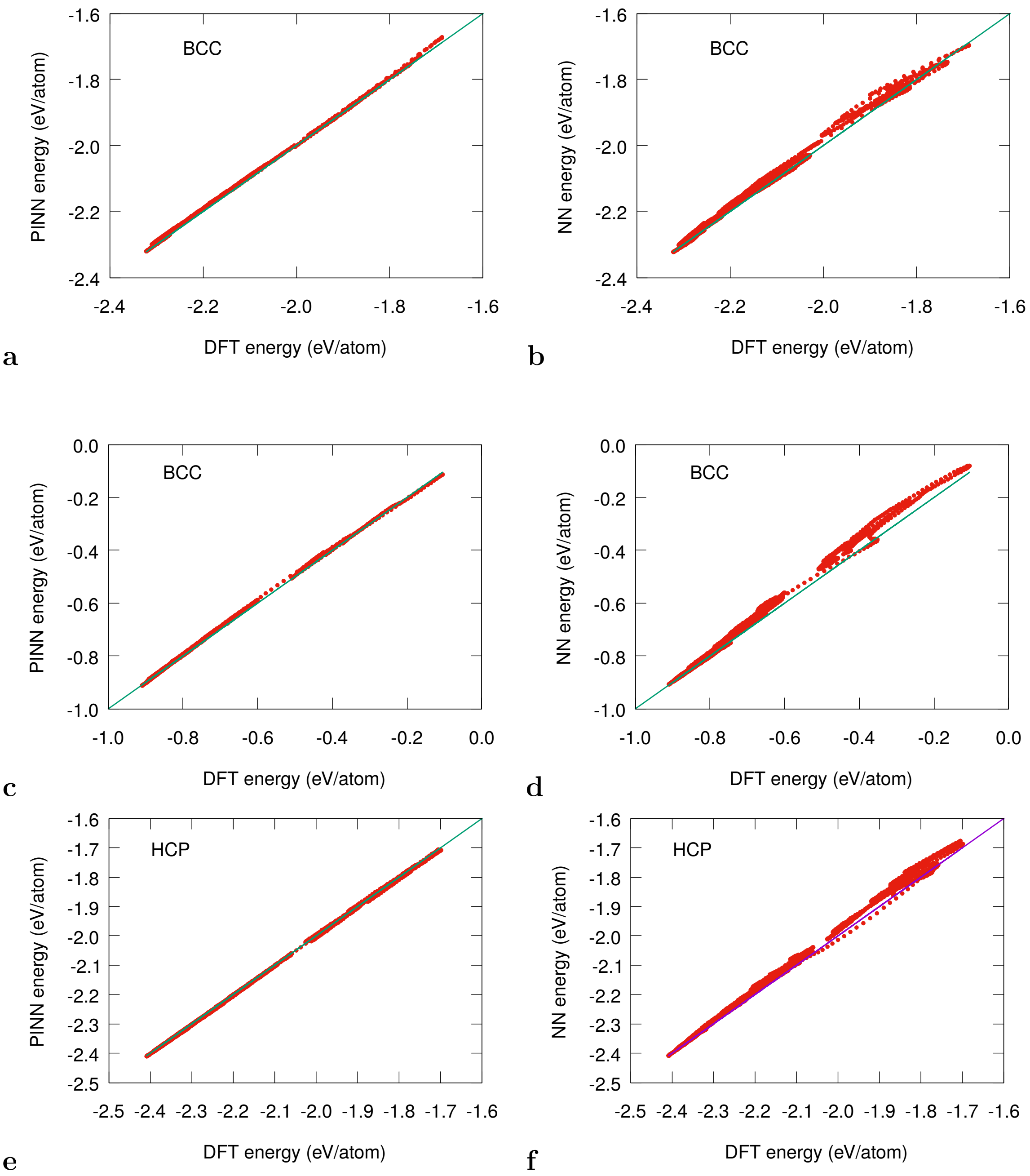

Supplementary Fig. 7 Energy of BCC and HCP Al in NVT MD simulations at the temperatures of (**a**, **b**, **e**, **f**) 300 K and 600 K and (**c**, **d**) 1000 K, 1500 K, 2000 K and 4000 K. The energies predicted by the PINN (**a**, **c**, **e**) and NN (**b**, **d**, **f**) potentials are compared with DFT calculations from [1, 2]. The straight lines represent the perfect fit.

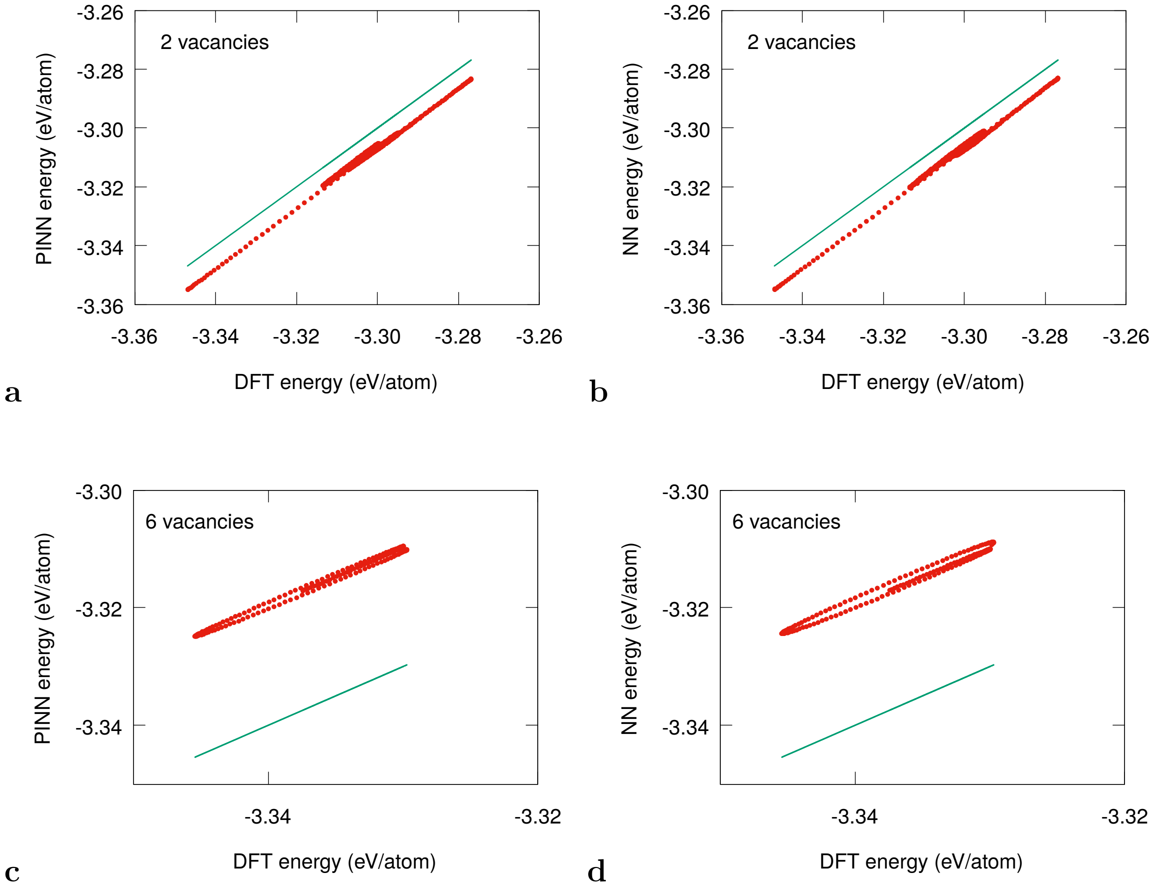

Supplementary Fig. 8 Energy of Al supercells containing (**a**, **b**) 2 and (**c**, **d**) 6 vacancies in NVE MD simulations starting at 700 K. The energies predicted by the PINN (**a**, **c**) and NN (**b**, **d**) potentials are compared with DFT calculations from [1, 2]. The straight lines represent the perfect fit.

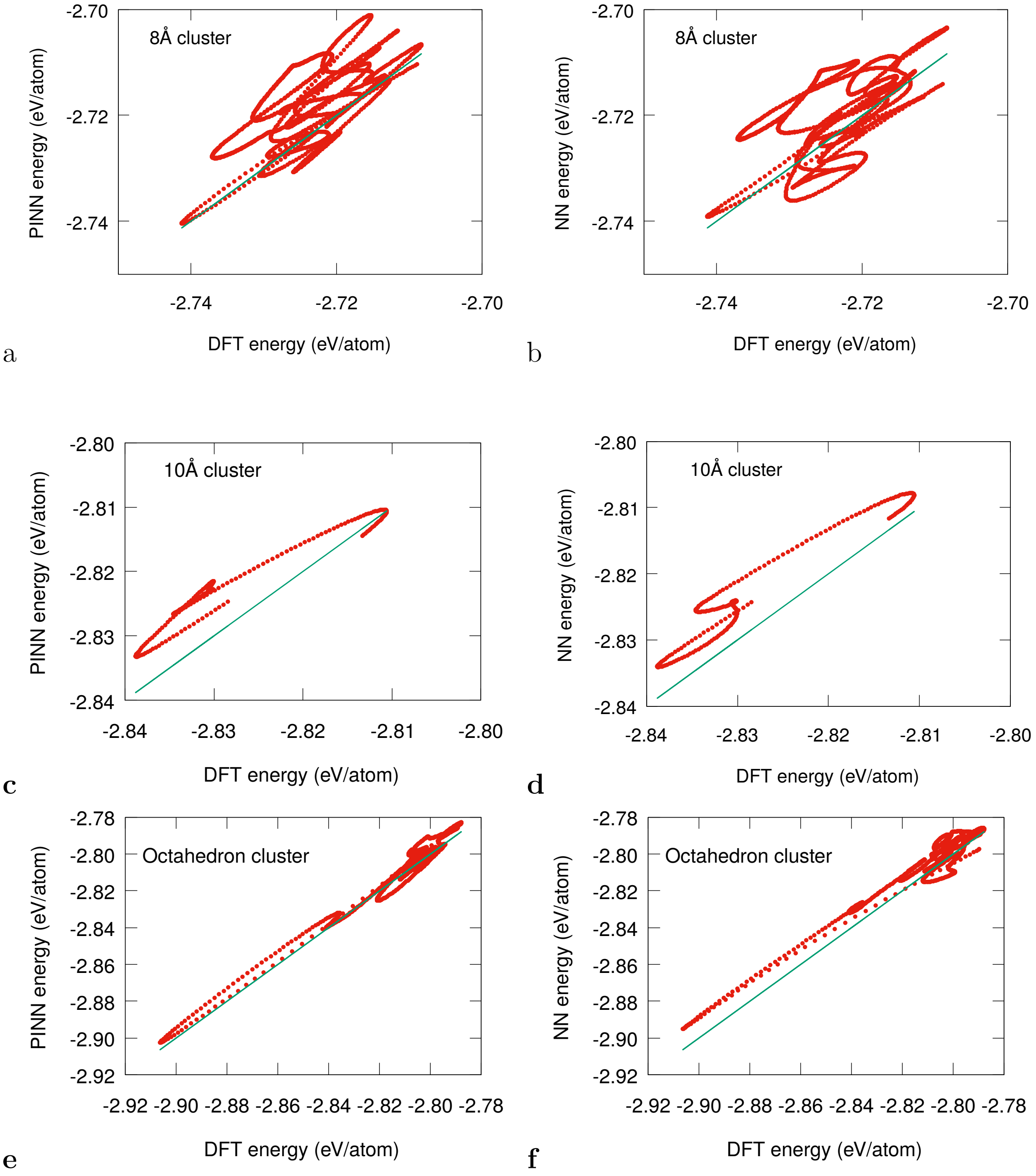

Supplementary Fig. 9 Energy of the 8 Å (a,b), 10 Å (c,d) and octahedral Al clusters in NVE MD simulations at the temperatures of (**a-d**) 1200 K and (**e**, **f**) 1000 K. The energies predicted by the PINN (**a**, **c**, **e**) and NN (**b**, **d**, **f**) potentials are compared with DFT calculations from [1, 2]. The straight lines represent the perfect fit.

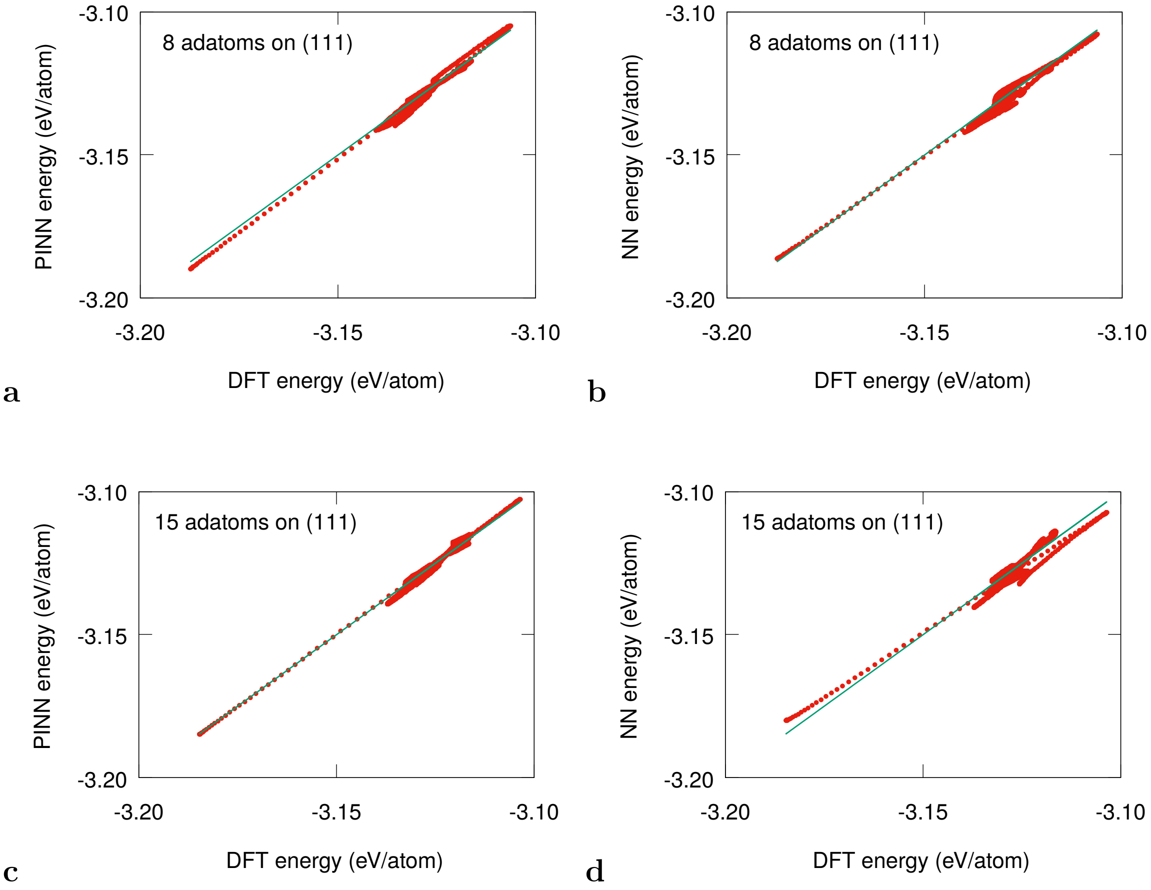

Supplementary Fig. 10 Energy of Al supercells containing (**a**, **b**) 8 and (c, d) 15 adatoms on the (111)FCC surface in NVE MD simulations starting at 1500 K. The energies predicted by the PINN (**a**, **c**) and NN (**b**, **d**) potentials are compared with DFT calculations from [1, 2]. The straight lines represent the perfect fit.

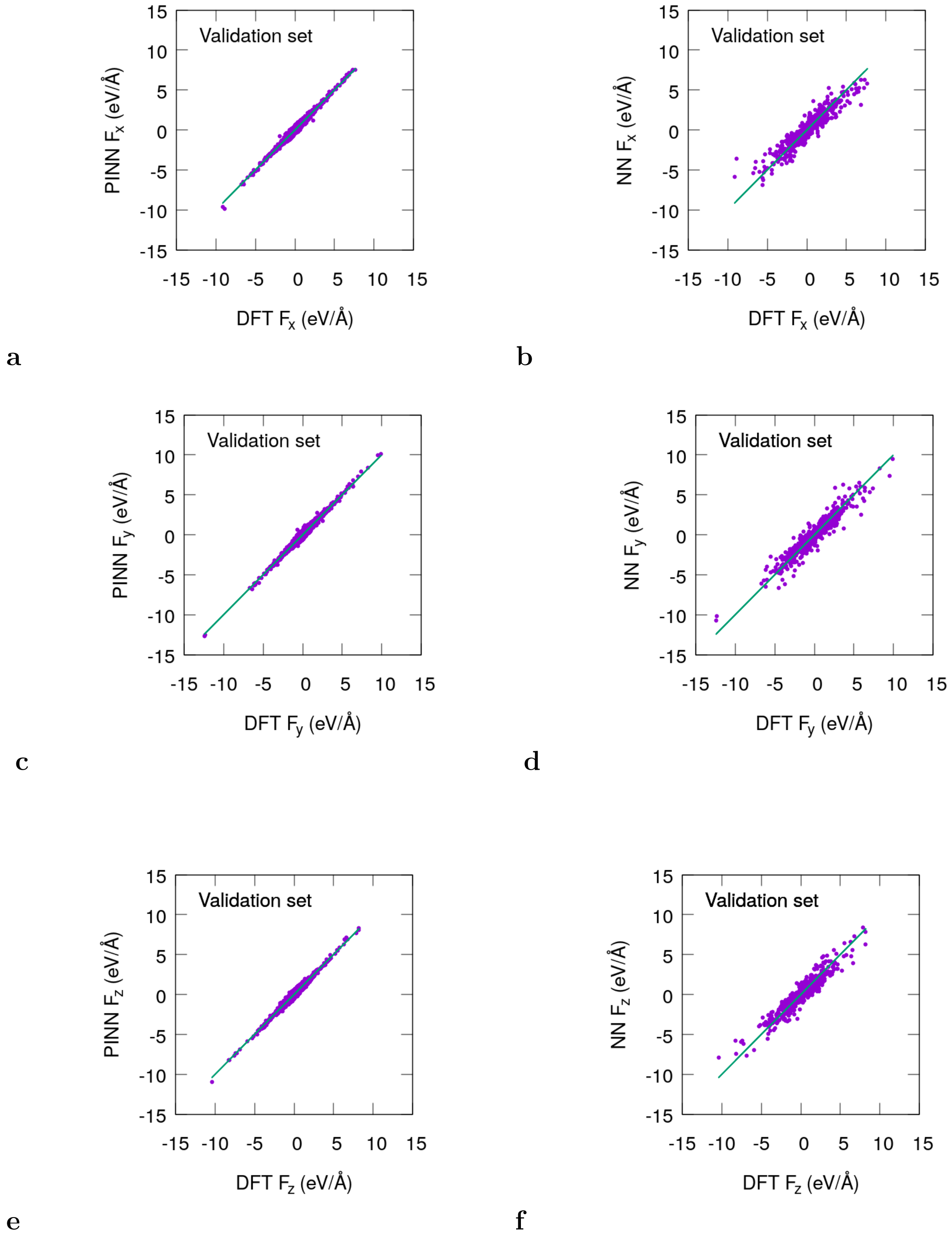

Supplementary Fig. 11 Atomic force components in validation database predicted by the PINN and NN potentials in comparison with with DFT calculations. The straight lines represent the perfect fit.

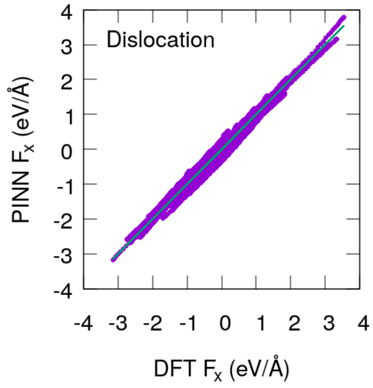

a

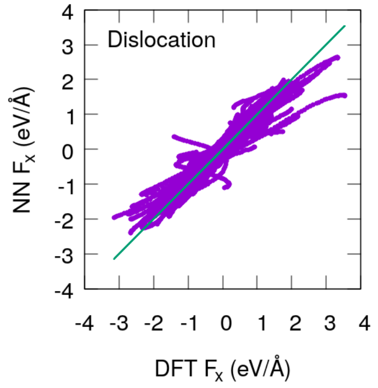

b

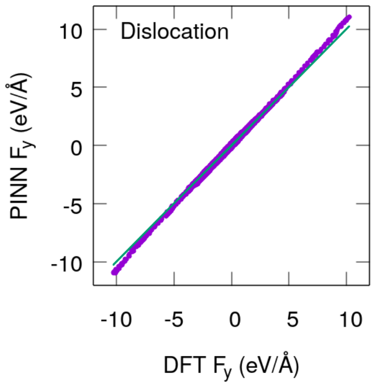

c

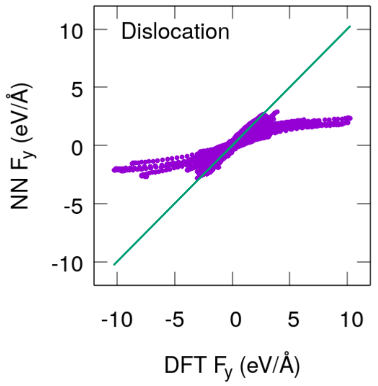

d

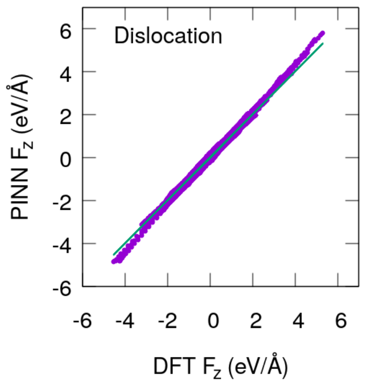

e

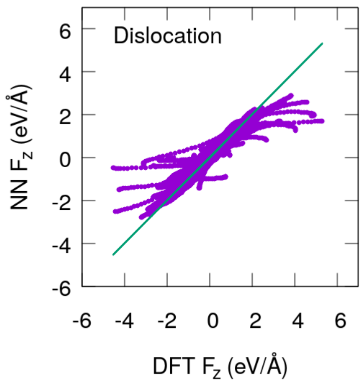

f

Supplementary Fig. 12 Atomic forces for the edge dislocation in NVE MD simulations starting at 700 K predicted by the PINN and NN potentials in comparison with DFT calculations. The straight lines represent the perfect fit.

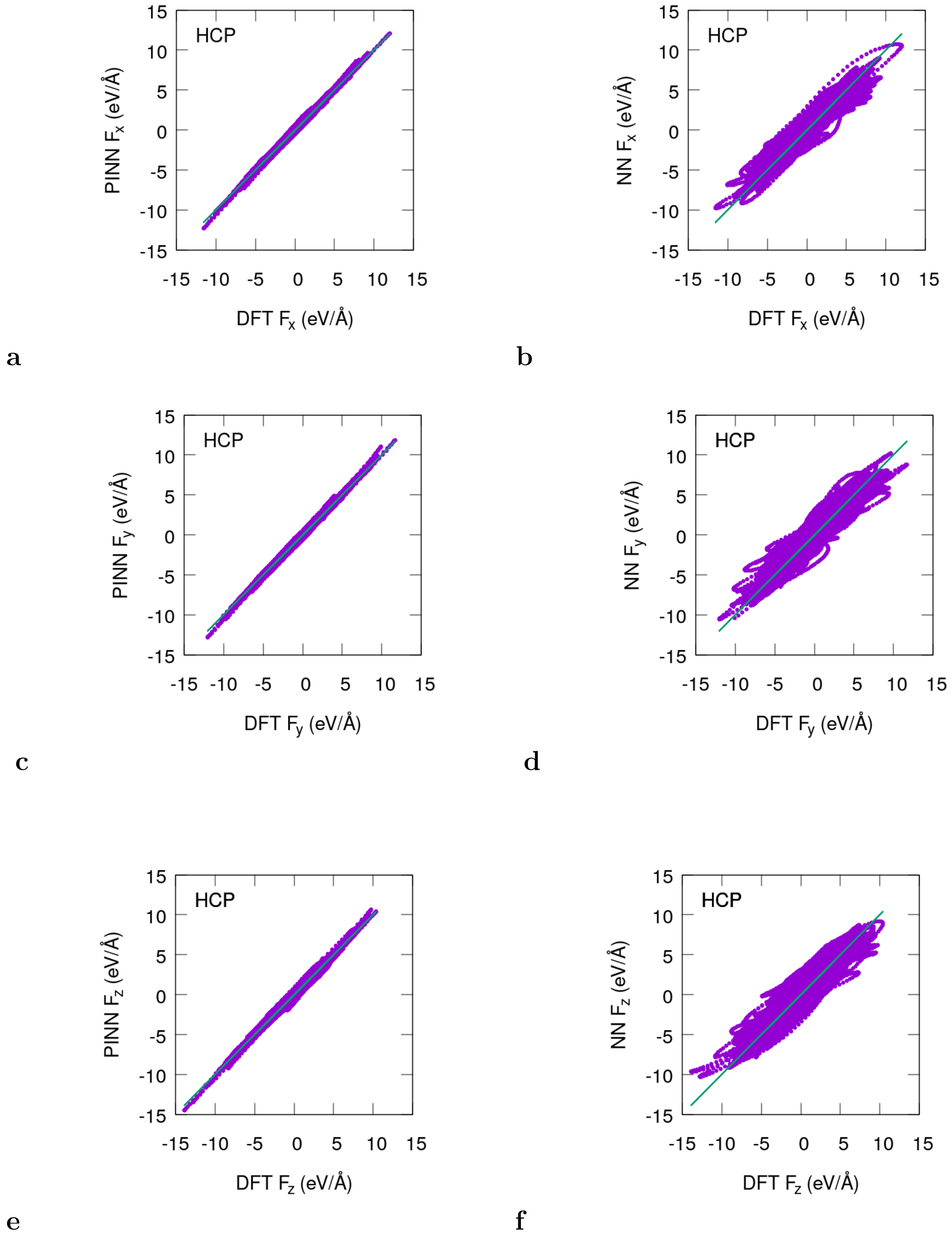

Supplementary Fig. 13 Atomic forces in HCP Al during NVT MD simulations at 300 K, 600 K, 1000 K, 1500 K, 2000 K and 4000 K predicted by the PINN and NN potentials in comparison with DFT calculations. The straight lines represent the perfect fit.

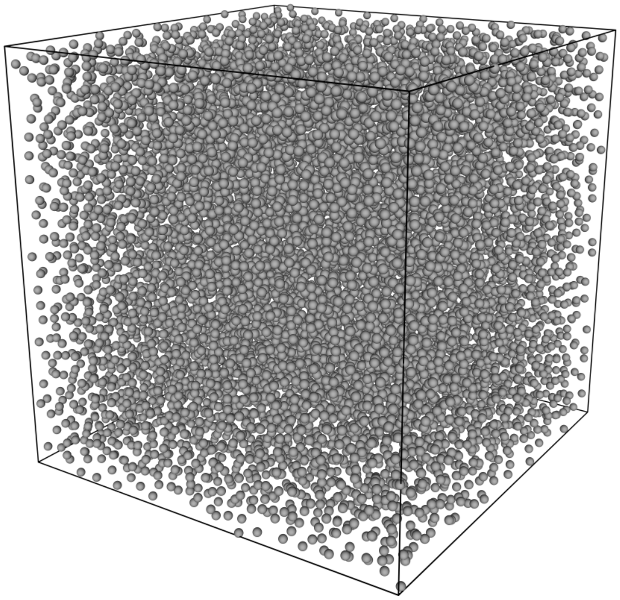

a

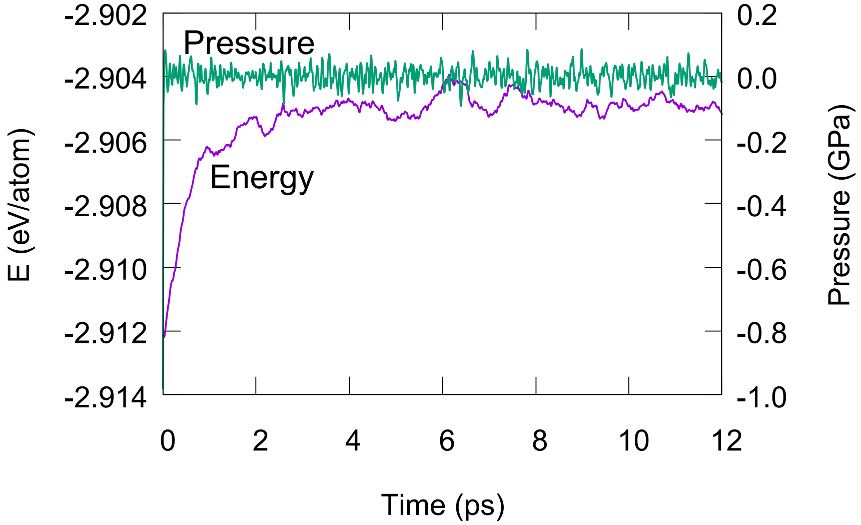

b

Supplementary Fig. 14 Demonstration of MD simulations for liquid Al with the PINN potential. The simulation was conducted in the zero-pressure NPT ensemble at the temperature of 1250 K using a beta-version of the ParaGrandMC code (https://software.nasa.gov/software/LAR-18773-1). The system contains 10,976 atoms. **a** Typical snapshot of the system. **b** Energy and pressure as a function of time during initial stages of the simulation.

Supplementary References

[1] Botu, V. & Ramprasad, R. Learning scheme to predict atomic forces and accelerate materials simulations. *Phys. Rev.* B **92**, 094306 (2015).

[2] Botu, V. & Ramprasad, R. Adaptive machine learning framework to accelerate ab initio molecular dynamics. *Int. J. Quant. Chem.* **115**, 1074–1083 (2015).

[3] Mauro, N. A., Bendert, J. C., Vogt, A. J., Gewin, J. M. & Kelton, K. F. High energy x-ray scattering studies of the local order in liquid Al. *J. Chem. Phys.* **135**, 044502 (2011).

[4] Jakse, N. & Pasturel, A. Liquid aluminum: Atomic diffusion and viscosity from ab initio molecular dynamics. *Scientific Reports* **3**, 3135 (2013).

[5] Alemany, M. M. G., Gallego, L. J. & González, D. J. Kohn-Sham ab initio molecular dynamics study of liquid Al near melting. *Phys. Rev.* B **70**, 134206 (2004).
